# Application of multiple genomic-editing technologies in *Streptomyces fungicidicus* for improved enduracidin yield

**DOI:** 10.1016/j.synbio.2025.02.008

**Published:** 2025-02-17

**Authors:** Yanan Sun, Guoguo Wu, Yining Wang, Jipeng Jiang, Haikuan Wang, Fufeng Liu, Fuping Lu, Huitu Zhang

**Affiliations:** Key Laboratory of Industrial Fermentation Microbiology, Ministry of Education, College of Biotechnology, Tianjin University of Science & Technology, Tianjin, 300457, PR China

**Keywords:** *Streptomyces fungicidicus*, Enduracidin, CRISPR/Cas, NRPS/PKS gene clusters deletion, Growth characteristics

## Abstract

*Streptomyces fungicidicus,* an industrial strain for enduracidin production, shows significant potential as a cellular chassis for the synthesis of novel small peptides. Targeted deletion of secondary metabolite gene clusters offers a promising strategy to enhance strain performance, but is often hampered by the lack of efficient gene editing tools. In this study, we optimized the traditional homologous recombination method by integrating selection and counter-selection markers to streamline the gene editing process, and successfully deleted gene clusters of up to 54.4 kb. Recognizing the significant potential of CRISPR/Cas-based systems in *Streptomyces*, we evaluated the base editing efficiency of the CRISPR/cBEST system in *S. fungicidicus*, which enabled stop codon insertions in the targeted gene with different mutation rates depending on the applied sgRNA. Additionally, we established a CRISPR/Cas9 system in *S. fungicidicus* while incorporating a counter-selection marker for efficient screening, which greatly shortened the gene editing cycle. The resulting mutants with single and cumulative gene cluster deletions exhibited improved growth characteristics, including a prolonged logarithmic phase and increased biomass. Although cumulative deletions did not result in consistent yield improvements, the mutants with improved growth characteristics show potential for further strain optimization in the future. The optimized gene editing systems developed in this study provide a valuable foundation for engineering other *Streptomyces* species.

## Highlights

• Optimized traditional HR method rapidly deleted a 54.4 kb gene cluster.
• Evaluated the base editing efficiency of the CRISPR/cBEST system.
• CRISPR/Cas9 system combined with counter-selection enabled efficient gene editing.
• Deletion of gene clusters improved strain growth characteristics.

## Graphical abstract

**Figure:**
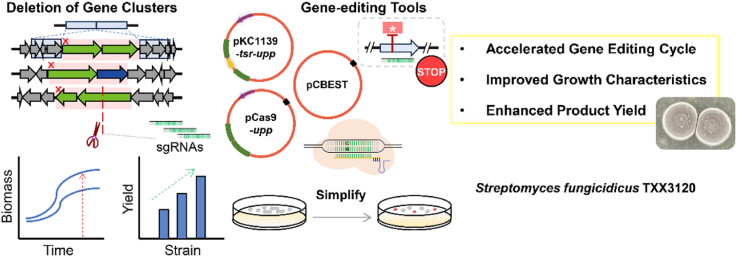

## 1 Introduction

Enduracidin, also known as enramycin, is a lipopeptide antibiotic produced by *Streptomyces fungicidicus*. It is a non-ribosomal peptide (NRP) with good biological activity and contains many specifically modified amino acid residues, such as the halogenated non-proteinogenic amino acid dichloro-hydroxyphenylglycine (Dpg), epimerized d-ornithine, and enduracididine (End) [1]. The biosynthetic gene cluster of enduracidin has been identified [2] and contains four genes encoding core non-ribosomal peptide synthetases (NRPS), which are involved in the recognition, activation, and assembly of 17 amino acid residues. This conforms to the typical linear assembly mechanism of substrates on NRPS, and has potential for modification to yield novel small peptides [3]. The genetic manipulation of *Streptomyces* is significantly more challenging than in model organisms such as *Escherichia coli* due to their complex genomic structure and slow growth rate.

Various technologies have been developed for genetic manipulation in *Streptomyces* species, including the traditional homologous recombination (HR) method [4], FLP/FRT recombination system [5], I-SceI endonuclease-based gene editing system [6,7], and CRISPR/Cas-based systems [[8], [9], [10]]. The double-strand break (DSB) generated by the Cas9 protein, followed by homology-directed repair (HDR) using a template, can achieve rapid and precise gene editing to streamline the screening process. The CRISPR/cBEST system, derived from the CRISPR/Cas system, is a cytosine base editor (CBE) fused to rat APOBEC-1 cytidine deaminase, nCas9 (D10A) protein and a uracil glycosylase inhibitor (UGI), which has been shown to achieve efficient base editing in several *Streptomyces* species [11,12]. Currently, several CRISPR systems have been developed and applied for editing the genome of *Streptomyces* [[13], [14], [15]]. However, the composition and expression of system components differ, and the efficiency of editing in different *Streptomyces* species has not been evaluated comparatively. Furthermore, there are significant differences in the genetic backgrounds of different *Streptomyces* strains, resulting in varying applicability and efficiency of gene editing tools in different strains [10,15,16]. Although several model *Streptomyces* species with clear genetic backgrounds and mature gene editing tools, such as *Streptomyces coelicolor* A3 [17], *Streptomyces albus* J1074 [18,19] and *Streptomyces lividans* TK24 [20], have been developed as chassis cells for heterologous expression, there are still many difficulties in heterologous expression of complete gene clusters, such as the challenging insertion of large fragments and inability to activate expression [16].

Enduracidin-producing *Streptomyces fungicidicus* is a non-model organism, in which genetic manipulation has traditionally relied on the HR method [21]. However, this approach involves multiple screening steps for single- and double-crossover recombination events. As a consequence, gene editing is a time-consuming and labor-intensive process in *Streptomyces* [4,5]. Recent advances in CRISPR/Cas9-based systems have demonstrated their potential for gene editing in *S. fungicidicus* ATCC21013 [22], but the development and application of novel gene editing tools in this species remains limited, which hinders efforts to improve production, strain development, and the discovery of new natural products. Therefore, the development of more suitable and efficient gene editing tools is crucial for the modification of *Streptomyce*s and the production of novel natural products. Studies on genetic resources reveal significant differences in the allocation of translation resources between essential and non-essential genes within the cell [23]. Genome simplification is a feasible strategy to construct ideal chassis strains, which generally entails deleting non-essential genes to reduce the metabolic burden and non-essential energy consumption [23], improve the growth characteristics of the strain [24], and promote the expression of heterologous genes [25,26]. Deletion of competing secondary metabolite biosynthetic gene clusters (BGCs) is an important strategy for improving strain performance [24]. In addition to increased yields [27], such mutants also exhibited various excellent properties, including a prolonged logarithmic phase [24], increased biomass [24,26], improved conversion rate [25], and genetic stability [26].

In this study, we present an optimized HR method incorporating selection and counter-selection markers, enabled the rapid deletion of a large gene cluster (up to 54.4 kb) with a shortened experimental period and fewer steps. We tested the feasibility and applicability of CRISPR/cBEST base editing in *Streptomyces fungicidicus*, and established a CRISPR/Cas9 gene editing system by integrating a counter-selection marker to improve editing efficiency. Using these approaches, we rationally constructed and systematically evaluated the mutants with targeted deletions of NRPS/PKS (polyketide synthase) gene clusters. This work has established a foundation for enhancing strain growth characteristics in *S. fungicidicus*, while providing a valuable reference for gene editing, genome streamlining, and yield improvement in other *Streptomyces* species.

## 2 Materials and methods

### 2.1 Strains, plasmids and reagents

All strains and plasmids used in this study are listed in Tables S1 and S2, respectively. *Streptomyces fungicidicus* TXX3120 (CICC11059) is an industrial enduracidin production strain, provided by Xinxing Veterinary Pharmaceutical Co. (Tianjin, China). The strain SF△*upp* was derived from *S. fungicidicus* TXX3120 by deletion of the endogenous background the uracil phosphoribosyl transferase gene (*upp*) gene, and was used as the strain to construct a series of recombinant strains in this study. *Bacillus subtilis* CMCC (B) 63501, used for detecting antibacterial activity of enduracidin, was purchased from the China Institute of Veterinary Drug Control (Beijing, China). *Escherichia coli* JM109 was used as the host for plasmid construction, while *E. coli* ET12567/pUZ8002 was used for intergeneric conjugation with *Streptomyces*. The plasmid pKC1139 was used for gene editing using the traditional HR method, pcBEST [28] was used to introduce genomic base changes in *Streptomyces*, while pCas9-*upp* was constructed based on the pcBEST backbone for gene editing.

The services of oligo synthesis (Table S3) and Sanger sequencing were provided by Genewiz (Tianjin, China). The ApexHF HS DNA polymerase (Accurate Biotech, Changsha, China), restriction enzymes (Takara, Dalian, China) and pEASY®-Basic Seamless Cloning and Assembly Kit (TransGen Biotech, Beijing, China) were used to amplify DNA fragments by PCR and plasmid construction. The digested DNA fragments from agarose gels and amplified DNA fragments from PCR reactions were isolated using the Gel Extraction Kit and Cycle Pure Kit (OMEGA, USA), respectively. Plasmid DNA was isolated from *E. coli* using the Plasmid Mini Kit (OMEGA). The enduracidin standard (4 %) was purchased from Yuanye Bio-Technology (Shanghai, China).

### 2.2 Media, culture conditions and transformation methods

*E. coli* strains were cultured at 37 °C on Luria-Bertani (LB; 10 g/L tryptone, 5 g/L yeast extract, 10 g/L NaCl) with the appropriate antibiotics. The procedures for plasmid extraction, transformation and preparation of competent cells were described in *Molecular Cloning:A Laboratory Manual* [29]. The strains were cultured using the insertion method for 4 days and cell morphology was observed using scanning electron microscopy (SEM).

*S. fungicidicus* TXX3120 and mutant strains were cultured at 28 °C on mannitol soya medium (MS; 20 g/L mannitol, 20 g/L soyabean flour and 20 g/L agar) for 7 days to induce sporulation. Seed cultures were obtained by transferring the resulting spores into 250 mL shake-flasks containing 50 mL of seed medium (22.4 g/L corn steep powder, 20 g/L CaCO_3_, 5.0 g/L cottonseed cake powder, 5 g/L (NH_4_)_2_SO_4_, 1.143 g/L FeSO_4_, 0.065 g/L KH_2_PO_4_, 35 g/L corn flour, pH 7.2), followed by incubation at 28 °C and 220 rpm for 48 h. The seed culture was then used to inoculate 500 mL shake-flasks containing 50 mL of fermentation medium (60 g/L corn flour, 5 g/L CaCO_3_, 24 g/L soybean flour, 2 g/L (NH_4_)_2_SO_4_, 1 g/L corn steep liquor, 5 g/L glucose, 0.1 g/L FeSO_4_, 1 mL/L lactic acid, 0.7 g/L thermostable α-amylase, pH 7.2) at a 10 % inoculation ratio, followed by incubation at 28 °C and 210 rpm for 10 days. Samples for analysis were taken every 12 h. Briefly, 1 mL of the culture broth was transferred into pre-weighed centrifuge tubes and centrifuged at 13,400 g‌ for 10 min. The supernatant was removed for pH measurement, and the increased weight of the centrifuge tube containing the pellet was recorded to determine the biomass. Three parallel replicates were included for each measurement.

The plasmids were transferred from the donor strain *E. coli* into the recipient strain *Streptomyces* by intergeneric conjugation as described before [4]. Briefly, *E. coli* ET12567/pUZ8002 was grown in 2 × YT medium (16 g/L tryptone, 10 g/L yeast extract, 5 g/L NaCl) at 37 °C with the appropriate antibiotics (50 mg/L apramycin, 25 mg/L chloramphenicol, and 50 mg/L kanamycin). When the OD600 reached 0.4–0.6, the culture was centrifuged and washed three times with precooled LB medium at 4 °C. The spores of *S. fungicidicus* were collected in 2 × YT medium and subjected to heat shock at 50 °C in a water bath for 10 min. Then, the spores were incubated at 28 °C for 3 h, and collected by centrifugation. MS medium with the addition of 10 mM MgCl_2_ was used for intergeneric conjugation between *E. coli* and *Streptomyces*. After 18 h of incubation, the mixture was covered with sterile water containing antibiotics (50 mg/L apramycin, 60 mg/L nalidixic acid), followed by incubation for 5–7 days to observe and verify the transformants.

### 2.3 Analytical methods for the assessment of enduracidin production

Enduracidin production can be detected by preliminary determination using the agar block method, semi-quantitatively using the filter paper method, and quantitatively using the high-performance liquid chromatography (HPLC). Enduracidin produced by *S. fungicidicus* fermentation generally accumulates intracellularly, so the precipitate of the fermentation broth must to be collected for extraction of enduracidin. To extract enduracidin for semi-quantitative and quantitative assays, 1.0 mL of the fermentation broth was centrifuged to collect precipitate, and 2.0 mL of the extraction solution (acetone:1 M HCl:ddH_2_O = 35:12:56), followed by extraction for 3.5 h. After centrifugation, the supernatant was filtered through a 0.22 μm pore-size organic membrane for subsequent analysis. Both the agar block method and filter paper method require the preparation of antibiotic test plates (2 % Antibiotic Assay Medium Ⅰ, Qingdao Hope Bio-Technology Co., Ltd., China, with 0.1 % spore suspension of *B. subtilis* CMCC 63501 on the upper layer, the lower layer is 2 % agar). The antimicrobial activity of the recombinant strains was initially detected using agar block method.

For preliminary determination, the mutants were cultured on maltose medium (8 g/L maltose, 1 g/L tryptone, 0.8 g/L yeast extract, 0.8 g/L tuna paste, 2.2 g/L agar powder) for 6 days, sliced into fixed size blocks and placed on the antibiotic test plates for 18 h.

For the semi-quantitative assay, the 6 μL of the extract supernatant was added to a sterile filter paper of 6 mm diameter, dried and carefully transferred to the antibiotic test plate. At the same time, an enduracidin standard was prepared using the same extraction method and diluted to different concentrations for control. The amount of enduracidin was calculated by measuring the diameter of the inhibition zone.

For the quantitative assay, HPLC was performed on an Agilent 1260 Infinity II instrument (Agilent Technologies, USA), equipped with an Agilent Eclipse XDB-C18 column (250 × 4.6 mm, 5 μm). The injection volume was 10 μL. The mobile phase was composed of 30 % buffer A (acetonitrile) and 70 % buffer B (50 mM NaH_2_PO_4_, pH 4.5) at a flow rate of 1.0 mL/min. Elution was monitored at 267 nm and quantified by comparing the peak arias with the standard curve.

### 2.4 Construction of pKC1139-based plasmids

The plasmid pKC1139-*upp*, derived from the plasmid pKC1139, contains a counter-selection marker, the *upp* gene, which was used for subsequent plasmid construction. The primers used in this study are listed in Table S3. The construction of the pKC1139-*upp*-derived plasmids was conducted as follows. The plasmid pKC1139-*upp* was digested with *Eco*RI and *Bam*HI restriction enzymes and the cut band recovered.

Using pKC1139-*upp*-t*nrps*3 as an example, the upstream fragments, amplified from the genome of *S. fungicidicus* TXX3120, were obtained using the primer pair nrps3-LF/nrps3-LR. The fragment was inserted into the pMD19-T vector (Takara), digested with *Bam*HI and *Xba*I restriction enzymes and purified for use. The downstream fragment was obtained using the primer pair nrps3-RF/nrps3-RR, inserted into the pMD19-T vector, and purified for use after digestion with *Eco*RI and *Xba*I. The up and downstream fragments were inserted into the *Eco*RI and *Bam*HI sites of the plasmid pKC1139-*upp*, resulting in pKC1139-*upp*-*nrps*3 (Fig. 2A), which contains only the homologous arms. The plasmid pKC1139-*upp*-*nrps*3 was digested with *Xba*I and then purified for use. The thiostrepton resistance gene (*tsr*) cassette was amplified from the plasmid pGH-*tsr* using the primer pair nrps3tsrF/nrps3tsrR, and then ligated into the linearized plasmid pKC1139-*upp*-*nrps*3 via the *Xba*I site. The plasmid containing the *tsr* selection marker used to delete of the *nrps*3 gene cluster was named pKC1139-*upp*-t*nrps*3 (Fig. 2A). Analogous procedures were used to obtain other plasmids, all of which were finally verified by Sanger sequencing.

### 2.5 Construction of mutant strains based on pKC1139 using selection and counter-selection markers

The pKC1139-*upp*-t*nrps*3 plasmid was introduced into strain SF△*upp* by intergeneric conjugation. Transformants were selected directly on MS plates containing apramycin at 40 °C for 4 days. The validated single-crossover colonies were then passaged onto MS plates containing thiostrepton and 5-fluorouracil for further culture at 28 °C for 4 days. The recombinant strains with successful deletion of the *nrps*3 gene cluster grew on the counter-selection plates and were verified by PCR using the primer pair nrps3OUT-F/nrps3OUT-R on either side of the homologous arm, named SF3, followed by Sanger sequencing. Analogous procedures were used to obtain the other mutants.

To sequentially delete *nrps*2 and *nrps*3 in the same strain, the *tsr* selection maker inserted into the genome had to be deleted. To achieve this, pKC1139-*upp*-*nrps*3 from the previous step was introduced into strain SF3 by intergeneric conjugation. The transformants were passaged to MS plates containing apramycin at 40 °C for 4 days, after which the validated single-crossover strains were passaged on antibiotic-free MS plates and screened for double-crossover by further incubation at 28 °C for 4 days. The subculture was repeated several times to increase the probability of double crossover when necessary. The strain resulting from the removal of the *tsr* selection marker was named SF4. The plasmid pKC1139-*upp*-t*nrps*2 was reintroduced into strain SF4, followed by repeated screening as described above. The resulting recombinant strain was verified by PCR using the primer pairs nrps2OUT-F/nrps2OUT-R and nrps3OUT-F/nrps3OUT-R.

### 2.6 Construction of pcBEST and pCas9-based plasmids

The plasmid pCRISPR–cBEST (Addgene 125689), which can convert C/G pairs in to T/A pairs by the guidance of sgRNA, contains an nCas9 (D10A) under the control of the thiostrepton inducible *PtipA* promoter, and an sgRNA cassette under the control of the *PermE∗* promoter. Prior to use, it only needs to be digested with *Nco*I restriction enzyme and purified to insert the sgRNA sequence. Because the effects of using Cas9 and nCas9 proteins in gene editing differ significantly among various *Streptomyces* species, we constructed two plasmids respectively encoding Cas9 and nCas9 proteins to explore their effects. The plasmid pCas9-*upp*, derived from pCBEST, deleted the genes coding for the fusion protein of cytidine deaminase and uracil glycosylase, replaced the nCas9 (D10A) coding gene with the Cas9 coding gene, and added a counter-selection marker downstream of the Cas9. Additionally, the *Xba*I restriction site for homologous template insertion was retained. The difference between the plasmids pnCas9-*upp* and pCas9-*upp* is that the Cas9 protein has not been replaced, and nCas9 (D10A) is still active, albeit with single-strand cleavage activity only. The relevant primers for the construction of plasmid pCas9-*upp* are listed in Table S3. For use, the plasmid pCas9-*upp* was first digested with *Xba*I purified to insert the homologous template. After verification by Sanger sequencing, it was digested with *Nco*I to insert the sgRNA sequence. The homologous template fragments were amplified from the genome of *S. fungicidicus* TXX3120 using the primers listed in Table S3. The sgRNAs were predicted using the CRISPy-web online server (https://crispy.secondarymetabolites.org/) [30]. Primers containing the sgRNA part were synthesized and annealed to obtain dsDNA for constructing plasmids.

### 2.7 Exploring the effects of base editing using pcBEST in *S. fungicidicus*

The original strain TXX3120 without gene editing was used as the test strain and the *upp* gene was the target gene to test the base editing effect of the cBEST system. We used the CRISPy-web online server [30] to design sgRNAs for CRISPR-BEST application. Seven sgRNAs were selected for the experiment, six of which were predicted to insert stop codons, and their location are shown in Fig. 3A. The details are shown in Table S6.

The plasmids pcBEST-based were introduced into strain TXX3120 by intergeneric conjugation. The mutants with a stop codon inserted in the *upp* gene were unable to grow in the presence of 5-fluorouracil. To investigate the mutation rate, the mutants were cultured in medium containing apramycin and 5-fluorouracil, respectively. The transformants with successful plasmid insertion could be grown normally on medium containing apramycin, but only the mutants with successful insertion of a stop codon in the *upp* gene could be grown on medium containing 5-fluorouracil. We collected the transformants from the conjugation plates, diluted them 10^7^–10^8^ fold, and then spread them on solid medium containing apramycin and 5-fluorouracil, respectively. The colonies were counted after incubating the plates at 28 °C for 4 days. Then, we continued to collect mutant strains grown on medium containing apramycin for dilution experiments to calculate the mutation rate at different numbers of passages for four generations. At least 6 mutants grown on medium containing 5-fluorouracil were randomly selected for sequencing in each generation. The conjugation efficiency was defined as the ratio of the number of transformants grown on conjugation plates to the number of recipient bacteria.

### 2.8 Construction of mutant strains using the pnCas9-*upp*/pCas9-*upp* plasmids

The plasmids pCas9-*upp* and pnCas9-*upp* require the insertion of a homologous repair template prior to use. After verification by Sanger sequencing, the corresponding sgRNA was inserted into the plasmid.

To investigate the gene editing effects of Cas9 and nCas9 proteins in *S. fungicidicus*, an independent type II thioesterase gene (CNQ36_25620, 825 bp) within the enduracidin gene cluster was selected as the target (Fig. 4A). The primers used for plasmid construction are listed in Table S3, while the construction and transformation procedures followed the methods described earlier. Transformants growing on conjugation plates were verified by PCR with the primers flanking the homologous arm and confirmed through Sanger sequencing. The correct recombinant strains were subsequently passaged on MS plates containing 5-fluorouracil at 40 °C for 5 days to cure the plasmid, resulting in the strain SF△*upp*△*endT*. When pCas9-*upp*-based plasmids containing only the sgRNA, without a homologous repair template, were introduced into strain SF△*upp* via conjugation, no transformants were obtained in multiple attempts. This suggests that HDR is the primary mechanism of DNA repair in *S. fungicidicus*, further limiting the likelihood of random mutations. Analogous procedures were performed to obtain SF6 and SF7 (Table S1).

## 3 Results

### 3.1 Analysis of BGCs and determination of deletion targets in *S. fungicidicus* TXX3120

**Fig. 1:**
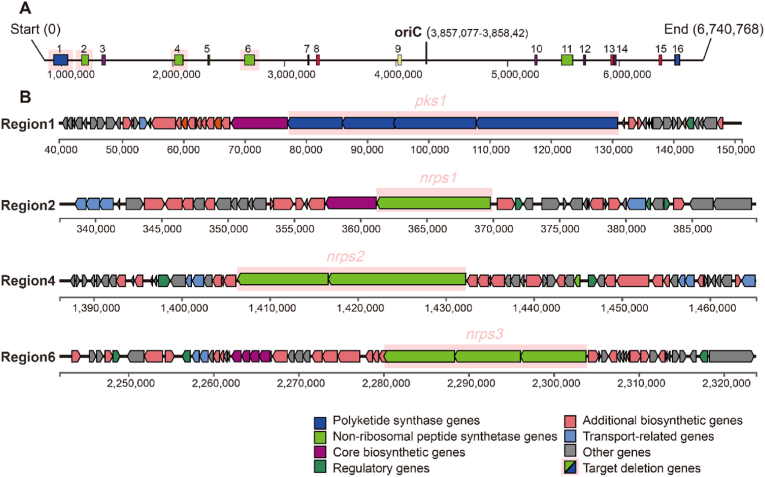
Analysis of the *Streptomyces fungicidicus* genome*.***(A)** Map of secondary metabolite biosynthetic gene clusters (BGCs) in the genome. The distribution of the 16 BGCs across the genome, and targeted deletion regions of BGCs are marked with light red shadow. **(B)** Details of the four targeted BGCs. Gene functions are colored as indicated in the legend. The targeted deletion regions are shaded in light red,which are polyketide synthetase (PKS) genes in region 1, and non-ribosomal peptide synthetase (NRPS) genes in region 2, 4, and 6.

The genome of *S. fungicidicus* TXX3120 has a length of 6,740,768 bp, containing 16 secondary metabolite BGCs predicted by antiSMASH Bacterial 6.0.1 [31], including NRPS, PKS, terpene, NI-siderophore, and RiPP-like BGCs (Fig. 1A). The details of the BGCs predicted by antiSMASH are listed in Table S4.

**Fig. 2:**
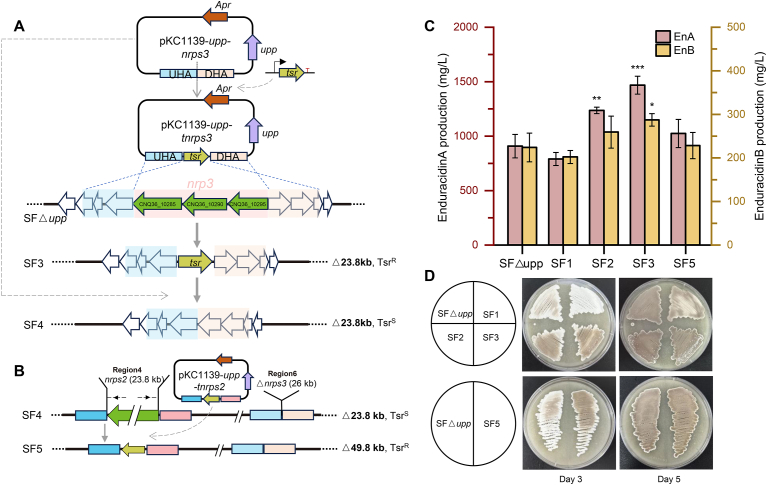
Construction of the mutants using the combined selection and counter-selection system. **(A)** Schematic diagrams of the construction process of SF3 and SF4. Construction of pKC1139-*upp*-*nrps* contains the apramycin resistance gene (*apr*orange arrow), the uracil phosphoribosyl transferase gene (*upp*) used as counter-selection screening marker (purple arrow), the upstream homology arm (UHA, pink shadow), and the downstream homology arm (DHA, blue shadow). pKC1139-*upp*-t*nrps* additionally contains an thiostrepton resistance gene (*tsr*, greenish-yellow arrow) as a selection marker between the homology arms. The corresponding plasmids were introduced into the strains by conjugation. The construction of SF1 and SF2 is similar to SF3. **(B)** Schematic diagrams of the construction process of SF5. **(C)** Enduracidin production (A and B) of the mutants and SF△*upp*. Data are presented as mean values ± SD. n = 3 biologically independent samples. P values were determined by one-way ANOVA + LSD multiple-comparison test (control group: SF△*upp*). ∗p < 0.05; ∗∗p < 0.01; ∗∗∗p < 0.001, non-significant values were not marked. **(D)** Phenotypic changes of the mutants compared with SF△*upp* on the third and fifth days on MS medium.

**Fig. 3:**
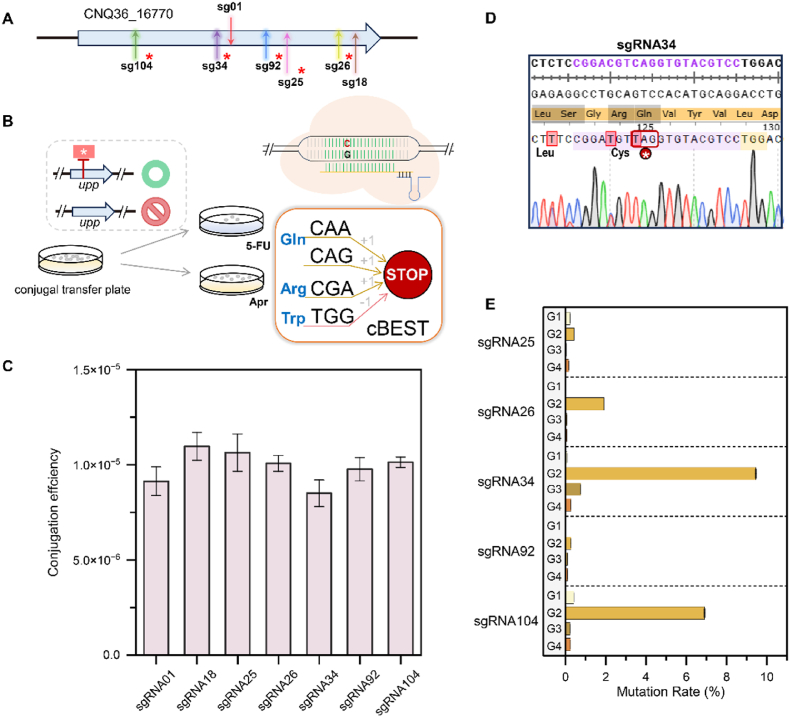
Base editing of the CRISPR/cBEST system in TXX3120 (WT) was performed using the *upp* gene (gene ID: CNQ36_16770) as the targeted gene. **(A)** The position of the designed sgRNAs in the *upp* gene. The sgRNAs predicted to insert stop codons were marked with red asterisks. The sgRNA01 is on the noncoding strand and all others are on the coding strand. **(B)** The mutants with a stop codon inserted into the *upp* gene could not grow on the 5FU-containing medium. To investigate the mutation rate, the transformants were subcultured on Apr-containing and 5FU-containing media. The codons encoding Gln (CAA, CAG), Arg (CGA), and Trp (TGG) would be mutated to stop codons by the CRISPR/cBEST system. **(C)** Conjugation efficiency of CRISPR/cBEST-based plasmids with different sgRNAs. **(D)** The sequencing result of the mutant with sgRNA34 inserted a stop codon. The editing window was 13 nt, and two amino acids were mutated (R124C, Q125∗). **(E)** The mutation rate of the mutants with different sgRNAs at different passage numbers.

**Fig. 4:**
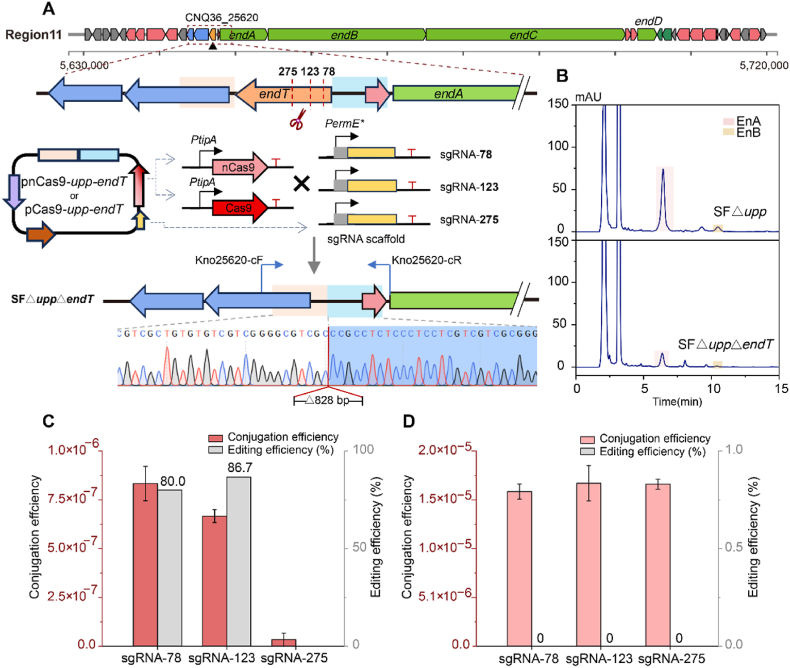
Gene editing of the CRISPR/Cas9 system in SF△*upp*, where the thioesterase gene of the enduracidin gene cluster (*endT*, gene ID: CNQ36_25620) was used as the targeted gene. **(A)** Three sgRNAs were designed targeting the *endT* gene. Construction of pCas9-*upp*-*endT* with the *apr* gene (orange arrow), the *upp* gene (purple arrow), UHA (pink shadow), DHA (blue shadow), Cas9 was under the control of the *PtipA* inducible promoter (red arrow), and the sgRNA scaffold cassette was under the control of the *PermE∗* constitutive promoter (yellow arrow). The difference in pnCas9-*upp*-*endT* was that the Cas9 was replaced with the nCas9 (D10A), which has only single-strand cleavage activity. The mutant SF△*upp*△*endT* successfully deleted the *endT* gene. **(B)** HPLC analysis of fermentation extracts from the control strain SF△*upp* and the mutant SF△*upp*△*endT* at λ = 267 nm. The peaks shaded in pink and yellow represent enduracidin A and B, respectivrly. **(C)** Conjugation efficiency and editing efficiency of pCas9-*upp*-*endT*-based plasmids with different sgRNAs. **(D)** Conjugation efficiency and editing efficiency of pnCas9-*upp*-*endT*-based plasmids with different sgRNAs.

In addition to the enduracidin gene cluster, there were three NRPS or NRPS-like BGCs. These targeted gene clusters for deletion had 100 % similarity to the known BGC alignment, producing antimycin, hormaomycin∗ and WS9326∗ (Fig. 1 and Table S4), respectively. The mechanism of PKS and NRPS synthesis involves successive condensation steps that link activated monomers into complex small-molecule compounds in an assembly-line principle. That polyketide metabolite gene cluster includes an extensive core biosynthetic gene cluster spanning up to 54.4 kb, which was identified as a target for deletion. This gene cluster shares 85 % similarity with the known BGC of dumulmycin and is located between positions 39,656 and 151,177 in the genome of TXX3120, as depicted in Region 1 of Fig. 1B.

Secondary metabolites of *Streptomyces* are non-essential for survival, while their gene clusters are typically large and consume significant energy during cellular growth, metabolism, and genome replication [20]. Rational genome reduction has been proposed as a viable strategy for constructing optimized chassis cells [26,32]. The monomers of NRPS assembly are both proteinogenic and non-proteinogenic amino acids, potentially leading to precursor competition between the enduracidin gene cluster and other NRPS clusters [24]. To address this, only the core NRPS/PKS genes were deleted, disrupting biosynthetic capabilities while retaining upstream synthesis genes to ensure a continued supply of precursors [25]. The targeted deletion of secondary metabolite gene clusters, including three NRPS and one PKS type, is depicted in Fig. 1B, with additional details provided in Table S5. The expression levels of the core genes of these BGCs are shown in Table S8.

### 3.2 Deletion of the PKS/NRPS core biosynthetic genes using the combined selection and counter-selection system

Genome editing of *S. fungicidicus* has traditionally relied on the HR method, which depends on natural single- and double-crossover events during passaging. However, the HR method is often inefficient, particularly for large deletions, due to constraints related to gene location and chromosomal structure [16]. To overcome these limitations, we developed a combined selection and counter-selection system using the *upp* gene as a counter-selection marker and the *tsr* gene as a selection marker.

The *upp* gene encodes uracil phosphoribosyl transferase (UPRT), which catalyzes the conversion of 5-fluorouracil (5-FU) into the toxic 5-fluorouridine monophosphate (5-FUMP). This direct and simpler approach bypasses the need for 5-fluorocytosine (5-FC) conversion as required by cytosine deaminase (codA) [8]. By constructing a null mutant of *upp* (CNQ36_16770) in *S. fungicidicus* TXX3120, we established an effective counter-selection system. However, challenges such as a high revertant mutation rate and difficulties in achieving doublecrossovers for large deletions persist. The introduction of *tsr* between homology arms can help avoid revertant mutations in strains where double crossovers have occurred. However, the use of the *tsr* selection marker alone does not facilitate the screening of the mutants with double-crossover events. Therefore, using the *tsr* selection marker in combination with the counter-selection system can significantly improve editing efficiency and reliability. This approach simplifies the gene editing process and prevents revertant mutations.

Using this system, we successfully deleted the core biosynthetic genes of PKS and NRPS clusters, resulting in the strains SF1, SF2, and SF3 with deletion sizes of 54.4 kb, 26.0 kb, and 23.8 kb, respectively (Fig. 2A and Table S5). Strain SF3 exhibited improved antibacterial activity compared to SF2, prompting the removal of *tsr* from SF3 to construct strain SF4, which served as the basis for further deletion of *nrps*2 (26.0 kb) to create SF5, with a cumulative deletion of 49.8 kb (Fig. 2B and Table S5).

Fermentation analysis revealed significant increases of enduracidin production in SF3 and SF2, with yields respectively improving by 61.6 % and 36.1 %, respectively, compared to the original strain SF△*upp* (Fig. 2C). By contrast, SF1 with a deletion of the *pks1* core gene showed a slight decrease in enduracidin yield (−12.9 %) and biomass, as well as delayed spore maturation [33] (Fig. 2D–S1B, and S1C). However, cumulative deletions in SF5 did not enhance enduracidin production further, reverting the yield to that of SF△*upp* (Fig. 2C). Nevertheless, SF2, SF3, and SF5 exhibited accelerated growth and spore maturation, with gray spores appearing on MS solid medium by the third day (Fig. 2D).

The deletion of core genes from non-essential PKS/NRPS gene clusters, which may compete for precursors and other intracellular resources, is a viable strategy to enhance the production of secondary metabolites [20,24]. By alleviating unnecessary metabolic burdens, such deletions can lead to improved growth rates [24,25] and create a more favorable metabolic environment, facilitating the efficient allocation of resources towards target biosynthetic pathways. This approach is particularly valuable for constructing chassis strains with simplified genetic backgrounds, optimized for synthetic biology applications [10,16,17].

The *tsr* marker proved effective for large fragment deletions but had limitations when constructing cumulative deletions due to the inherently slow growth of *Streptomyces*, which prolonged experimental timelines [16]. Thus, further advancements in efficient and streamlined genome-editing techniques are necessary to optimize strain engineering.

### 3.3 Application and evaluation of the CRISPR/cBEST system using the *upp* gene

The CRISPR/cBEST system has demonstrated efficient base editing in several *Streptomyces* species, particularly in the insertion of stop codons in genes with high GC content through targeted site selection [28]. This method bypasses homologous recombination and simplifies plasmid construction. To assess its applicability in *S. fungicidicus*, we used the original strain TXX3120 and targeted the *upp* gene (CNQ36_16770), which can be directly counter-selected with 5-fluorouracil.

Using CRISPy-web [30], we designed seven sgRNAs, six of which were predicted to insert stop codons (Fig. 3A–Table S6). The conjugation efficiency of plasmids carrying different sgRNAs was similar at approximately 1 × 10^−5^, but the mutation rate varied significantly among the sgRNAs (Fig. 3C and E). These results confirm that the location of the sgRNA target site is a critical factor influencing the editing efficiency of the CRISPR/cBEST system [28]. The mutation rate of the transformants varied greatly after different numbers of passages (Fig. 3E). For sake of brevity, here we used "sgRNAx" to designate mutants generated using the corresponding sgRNAs.

As expected, each generation of transformants using sgRNA01 was unable to grow on medium containing 5-fluorouracil, but unexpectedly, transformants using sgRNA18 also never grew on medium containing 5-fluorouracil. The highest mutation rate was observed in the second generation of mutants, with sgRNA34 showing a peak mutation rate of 9.2 % (Fig. 3E). However, no further increase of mutation rate was observed with subsequent passages.

In the sequencing results of the mutants, we observed single and multiple editing events for different sgRNAs. There were single editing events with sgRNA18, sgRNA92, and sgRNA104, and their editing windows were stable, although it was different from the CRISPy-web prediction. In the results of sgRNA25, we observed base editing from G to A at position −1 upstream of the PAM sequence. A similar result was observed with sgRNA26, with base editing from C to G at position −5 upstream of the PAM sequence. However, four kinds of editing events occurred in the mutants induced by sgRNA01 without introduction of stop codons. The maximum editing window in one editing event was 14 nt, and base editing could occur from −1 to 17 upstream of the PAM sequence in the mutants induced by sgRNA01. The amino acid substitution, V128I (GAC to GAT) predicted by CRISPy-web [30], achieved an editing efficiency of 100 % by converting C to T. Among the mutants induced by sgRNA34, the maximum editing window in one editing event was 11 nt, and the coding strand had changes from C to T at positions −3 to 8 upstream of the PAM sequence, the change of a Gln (CAG) to a STOP codon (TAG) reached an editing efficiency of 100 %. All sequencing results and peak maps are shown in Fig. S2.

Thus, we developed a rapid and efficient method for assessing the efficiency of stop codon insertion using the CRISPR/cBEST system in *S. fungicidicus* by targeting with *upp* gene. This assay was used to investigate the mutation rate of stop codon insertions using different sgRNAs across multiple passages, and sequencing of randomly selected clones reveals the diversity of editing events. The cytosine base editor consisting of APOBEC-1 inserts a stop codon on the chromosome of *S. fungicidicus* to block gene expression and can be used to probe gene function, but the instability of the editing window and editing events makes it unsuitable for precise base editing. The performance of the cytosine base editor varied considerably, while inefficient editing and editing effects influenced by the different sequence contexts of the target sites were also observed, in agreement with the studies in *S. lividans* 66 and *S. coelicolor* [34]. The editing window was found to be larger than previously reported in other *Streptomyces* species (from positions 4 to 8) [28,34], and extending upstream of the PAM sequence. While the preferred sequence context for APOBEC1 substrates is TC or CC [35,36], some relaxation in this preference was observed, resulting in a variety of editing events and expanded editing windows. The editing efficiency varied significantly across different sgRNAs, with some showing notably higher effectiveness than others. This finding emphasizes the importance of sequence selection, suggesting that testing multiple sgRNAs may be beneficial.

### 3.4 Construction and evaluation of the CRISPR/Cas9-based counter-selection system

The CRISPR/Cas-based gene editing has been widely used in various species of *Streptomyces* due to its accuracy and efficiency [14,37]. However, the cytotoxicity caused by Cas9-generated DSBs greatly reduced the conversion rate [32]. By contrast, nCas9 (D10A), which induces single-strand cleavage, avoids this shortcoming [15]. We first constructed two versions of gene editing systems, using the thiostrepton-inducible promoter *PtipA*, linked to Cas9 and nCas9 expression cassettes, respectively. The *upp* selection marker was included to cure the plasmid after editing.

We selected a type II thioesterase encoding gene, *endT* (CNQ36_25620), upstream of the core biosynthetic gene *endA* of the enduracidin gene cluster, as the target gene for testing, which is pivotal for enduracidin biosynthesis. We selected three sgRNAs designed by CRISPy-web [30], targeting *endT* at different sites, resulting in a total of six plasmids using Cas9 or nCas9 (Fig. 4A).

In the mutants produced using Cas9, the conjugation efficiency of sgRNA78 and sgRNA123 was much higher than that of sgRNA275, with editing efficiency of more than 80 % (Fig. 4C). After repeated experiments, we were still unable to screen successfully edited mutants from the transformants generated using sgRNA275. Curiously, primers on either side of the HAs consistently failed to amplify any fragments of either the original or successfully deleted size. By contrast, the conjugation efficiency of three sgRNA using nCas9 was about approximately 500 times higher than that of Cas9, but no successfully edited recombinant strains were screened among these transformants (Fig. 4D). HPLC analysis revealed that the production of enduracidin was significantly reduced in the mutant with deletion of *endT* (Fig. 4B).

The transformation efficiency of nCas9 was much higher than that of Cas9, indicating that the DSBs generated by Cas9 greatly reduce the conjugation efficiency, but maintain a high rate of successful editing [38]. A possible reason for this is that nCas9 only produces single-strand breaks, which are not sufficient to induce HDR repair in the strain. The position of sgRNA275 is located in the middle of *endT*, which is far from the HAs on both sides. This makes it difficult to repair the resulting DSB via the HDR pathway, which may be the reason why sgRNA275 using Cas9 had an extremely low conjugation efficiency with no successfully edited mutants using Cas9. The efficiency of gene editing is strongly influenced by the position of the sgRNA, with proximity to target or homology regions often being correlating with enhanced editing outcomes [38].

In short, the gene editing system of Cas9 with appropriate sgRNAs has a high gene editing efficiency in *S. fungicidicus*, which reduces the times of screening steps and shortens the gene editing cycle. Consequently, the plasmid pCas9-*upp* was selected as the gene editing tool for subsequent experiments.

### 3.5 Decline of enduracidin production following the sequential deletion of NRPS gene clusters

**Fig. 5:**
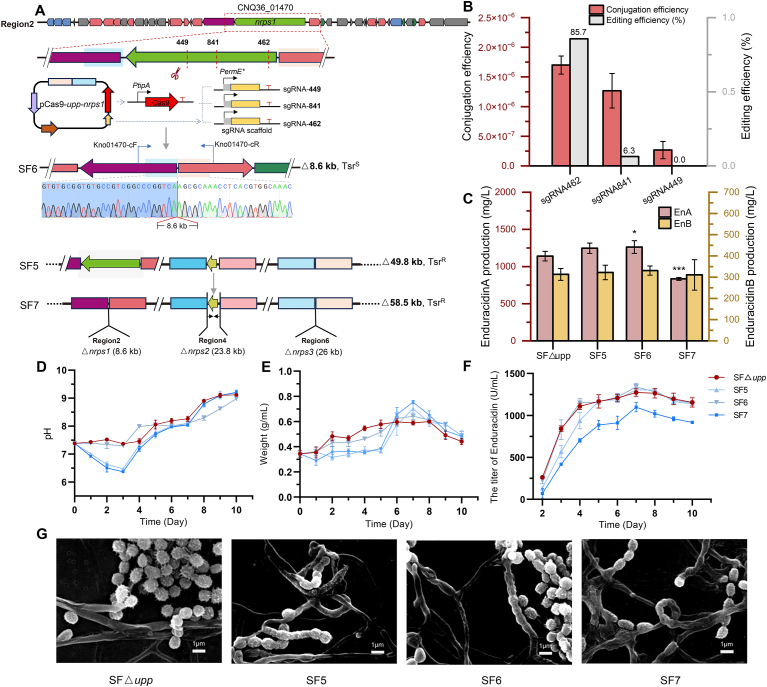
Application of the CRISPR/Cas9 system in SF△*upp*, where the NRPS gene of region 2 (gene ID: CNQ36_01470) was used as the target deletion gene. **(A)** Three sgRNAs were designed. The pCas9-*upp-nrps*1-based plasmids with three different sgRNAs were introduced into the strains by conjugation. SF6 was derived from SF△*upp* with deletion of the *nrps*1 gene, and SF7 was derived from SF5 with deletion of the *nrps*1 gene, resulting in a total deletion of 58.5 kb from three BGCs. **(B)** Conjugation and editing efficiency of CRISPR/nCas9-*upp-nrps*1-based plasmids with three different sgRNAs. **(C)** Enduracidin production (A and B) of the mutants and SF△*upp*. Data are presented as mean values ± SD. n = 3 biologically independent samples. P values were determined by one-way ANOVA + LSD multiple-comparison test (control group: SF△*upp*). ∗p < 0.05; ∗∗∗p < 0.001, non-significant values were not marked. **(D)** pH variation of the mutants and SF△*upp* during fermentation*.***(E)** The growth profiles of the mutants and SF△*upp* during fermentation. **(F)** The titer of enduracidin of the mutants and SF△*upp* during fermentation. **(G)** Cell morphology determination using the scanning electron microscopy (SEM) for the mutants and SF△*upp*.

The core biosynthetic gene of *nrps*1 in region 2 is 8,637 bp long, and has not been successfully deleted previously by traditional HR methods. We designed 1,200 bp homology arms up- and downstream of the target gene. Three sgRNAs with low off-target rates and different positions designed by CRISPy-web [30] were selected for *nrps*1 deletion (Fig. 5A). The conjugation efficiency of sgRNA462 was higher than that of sgRNA841 and sgRNA449. The editing efficiency of sgRNA462 was 85.7 %, and only 6.3 % of sgRNA841 was successfully edited (Fig. 5B). However, we were unable to screen successfully edited mutants induced by sgRNA449. The mutant with successful deletion of *nrps*1 based on SFA was named SF6, and the mutant with *nrp*s1 deletion based on SF5 was named SF7 (Fig. 5A).

An appropriate sgRNA is a critical factor for successful gene editing with Cas9 [37]. However, determining a universally effective principle remains challenging and requires extensive testing across various hosts and genes. Studies have shown that positioning the sgRNA closer to the homology arm enhances editing efficiency [38], likely due to improved repair of the Cas9-induced DSB via the HDR pathway [39,40].

The yield of enduracidin in the mutants differed from expectations. The strain SF6, in which only *nrps*1 was deleted, showed a 10.8 % increase of yield compared to strain SF△*upp.* However, strain SF7, which had three NRPS core genes deleted, exhibited a yield decrease of approximately 26.8 % (Fig. 5C).

Compared with SF△*upp*, strain SF6 showed similar pH and yield trends, with slightly lower biomass than SF△*upp* from days two to six, but slightly higher after day seven, showing a mild upward trend in the later stages. By contrast, the pH trend of strain SF7 was similar to that of its precursor strain SF5, initially decreasing to a minimum of 6.37 on day three and then increasing from day four. Its growth trend differed significantly from SF△*upp*, with low biomass observed in the first five days, followed by rapid growth on days five and six, reaching a peak on day seven. Throughout the entire fermentation cycle, the yield of SF7 remained lower than that of the other strains. Overall, the deletion of non-essential genes can have very complex effects on fermentation [41], not only affecting the yield of target metabolites but also causing fluctuations in the metabolic balance of the mutants, changing the pH and fermentation time [23].

The deletion of *nrps*1 influenced spore coloration, with strains SF6 and SF7 exhibiting lighter spore colors compared to other mutants. The *nrps*1 is part of an NRP-polyketide hybrid gene cluster, and spore coloration is linked to the products of polyketide biosynthesis pathways [33].

While genomic deletions may not consistently enhance the yield of target secondary metabolites, they do significantly impact the strain's metabolism and growth. Therefore, rational design and modification of strains are essential. Additionally, constructing industrial strains with streamlined and clearer metabolic backgrounds provides a strong foundation for their use as chassis cells [42]. Although sequential deletions of gene clusters do not guarantee a continuous increase of secondary metabolite production, strategic removal of non-essential genes can improve the strain's growth performance and extend the fermentation cycle [24,25].

## 4 Discussion and conclusion

In this study, we systematically investigated the effects of single and sequential deletions of NRPS and PKS gene clusters in *Streptomyces fungicidicus*. Our findings showed that deletion of a single gene cluster can improve the enduracidin yield, while sequential deletion cannot lead to a continuous increase in yield. Instead, multiple deletions can reduce production due to disruptions in the metabolic balance and energy distribution of the cells, even when growth characteristic are unaffected [43]. A possible explanation is the competitive and interdependent relationship between strain growth and secondary metabolism [44,45]. Rapidly growing strains tend to prioritize the synthesis of primary metabolites [46,47], such as nucleic acids and proteins, which may delay or reduce the production of secondary metabolites such as antibiotics [48,49].

Additionally, we observed that the deletion of non-essential genes affected the intracellular metabolic activities [50], while significantly altering fermentation parameters such as pH, biomass and fermentation time, which is attributed to the complexity of metabolic responses to genome reduction. Despite these challenges, the rational deletion of non-essential gene clusters improved the strain growth characteristic and prolonged the fermentation time, demonstrating its potential as a strategy for developing robust chassis cells suitable for industrial applications [42].

By combining traditional HR methods with a selection and counter-selection system, we achieved efficient large-fragment deletions, which significantly shortened the gene editing cycle. In addition, the CRISPR/cBEST system was successfully adapted for base editing in *S. fungicidicus*, but had a broader editing window with diverse editing events. By selecting suitable and effective sgRNAs, the CRISPR/Cas9 gene editing system with a counter-selection marker can rapidly and efficiently delete the genomic fragments in *Streptomyces fungicidicus*, significantly reducing experimental steps and improving gene editing efficiency. These results provide valuable insights for the optimization of gene editing tools.

Overall, our work has established a foundation for more effective engineering of *Streptomyces* strains. These findings advance the application of genome editing and synthetic biology in the development of industrially relevant microbial chassis cells, and provide a valuable reference for future strain optimization and enhanced production.

## CRediT authorship contribution statement

**Yanan Sun:** Writing – original draft, Visualization, Investigation, Formal analysis, Data curation. **Guoguo Wu:** Validation, Investigation, Formal analysis, Data curation. **Yining Wang:** Visualization, Validation, Formal analysis. **Jipeng Jiang:** Investigation, Formal analysis, Data curation. **Haikuan Wang:** Writing – review & editing, Supervision, Conceptualization. **Fufeng Liu:** Supervision, Formal analysis, Conceptualization. **Fuping Lu:** Supervision, Resources, Project administration, Conceptualization. **Huitu Zhang:** Writing – review & editing, Supervision, Resources, Project administration, Funding acquisition, Conceptualization.

## Ethics approval

This article does not contain any studies with human participants or experimental animals performed by any of the authors.

## Funding

This work was supported by the 10.13039/501100001809National Natural Science Foundation of China (NO. 82073743).

## Declaration of competing interest

The authors declare that they have no known competing financial interests or personal relationships that could have appeared to influence the work reported in this paper.
