# Supplementary material for: Application of multiple genomic-editing technologies in Streptomyces fungicidicus for improved enduracidin yield
Source: Synth Syst Biotechnol. 2025 Feb 17;10(2):564–73. doi: 10.1016/j.synbio.2025.02.008 (PMC11909717; doi:10.1016/j.synbio.2025.02.008)
Supplement: Multimedia component 1 [file mmc1.docx]

**Supplementary data**

**Deletion of gene clusters using a CRISPR/Cas9 system in *Streptomyces fungicidicus* and its effect on enduracidin biosynthesis and growth development**

Yanan Sun, Guoguo Wu, Yining Wang, Jipeng Jiang, Haikuan Wang, Fufeng Liu, Fuping Lu, Huitu Zhang^🖂^

**Author Affiliations**

Key Laboratory of Industrial Fermentation Microbiology, College of Biotechnology, Tianjin University of Science & Technology, Tianjin, 300457, PR China

**^🖂^ Corresponding author:** Huitu Zhang

**Mailing address:** No. 29, 13 Main Street, Tianjin Economic and Technological Development Area, Tianjin 300457, PR China

**Phone:** +86-22-60601958

**Fax:** +86-22-60600810

**E-mail:** [**hzhang@tust.edu.cn**](mailto:hzhang@tust.edu.cn)

**Table S1** The strains used in this study

| **Strains** | **Relevant characteristics** | **Sources** |
| --- | --- | --- |
| *E. coli* JM109 | Host for plasmid general cloning | Maintained in lab |
| *E. coli* ET12567/pUZ8002 | For conjugation transfer between *E. coli* and *Streptomyces* | Maintained in lab |
| *S. fungicidicus* TXX3120 | Wild type, industrial strain of enduracidin | Xinxing Veterinary Pharmaceutical Co.  (Tianjin, China). |
| *B. subtilis* CMCC (B) 63501 | Used for antimicrobial activity of enduracidin | Maintained in lab |
| SFΔ*upp* | Derivative of wild type *S.fungicidicus* TXX3120 with upp gene (CN136_16770) deletion | Maintained in lab |
| SF1 | Derivative of SFΔ*upp* without PKS1 | This work |
| SF2 | Derivative of SFΔ*upp* without NRPS2 | This work |
| SF3 | Derivative of SFΔ*upp* without NRPS3 | This work |
| SF4 | SF3 with removed the *tsr* selection marker | This work |
| SF5 | Derivative of SFΔ*upp* without NRPS2 and NRPS3 | This work |
| SF6 | Derivative of SFΔ*upp* without NRPS1 | This work |
| SF7 | Derivative of SFΔ*upp* without NRPS1, NRPS2 and NRPS3 | This work |
| SFΔ*upp*Δ*endT* | Derivative of SFΔ*upp* without *endT* gene (CN136_25620) | This work |
|  |  |  |

**Table S2** The plasmids used in this study

| **Plasmids** | **Relevant characteristics** | **Sources** |
| --- | --- | --- |
| pKC1139-*upp* | Derived from pKC1139, gene editing vector for traditional HR method with the *upp* counterselection marker | Maintained in lab |
| pGH-*tsr* | Vector containing the thiostreptothritin resistance gene (*tsr*) cassette | Maintained in lab |
| pKC1139-*upp-pks1* | pKC1139-*upp* containing two HAs flanking PKS1 | This work |
| pKC1139-*upp-*t*pks1* | pKC1139-*upp-pks1* containing the tsr selection marker | This work |
| pKC1139-*upp*-*nrps2* | pKC1139-*upp* containing two HAs flanking NRPS2 | This work |
| pKC1139-*upp-*t*nrps2* | pKC1139-*upp*-*nrps2* containing the tsr selection marker | This work |
| pKC1139-*upp*-*nrps3* | pKC1139-*upp* containing two HAs flanking NRPS3 | This work |
| pKC1139-*upp*-t*nrps3* | pKC1139-*upp*-*nrps3* containing the tsr selection marker | This work |
| pCRISPR-cBEST | Base editing vector containing cytidine deaminase base editor and sgRNA scaffold cassette, short for pcBEST |  |
| pcBEST-sgRNA01 | pCRISPR-cBEST with sgRNA01 | This work |
| pcBEST-sgRNA18 | pCRISPR-cBEST with sgRNA18 | This work |
| pcBEST-sgRNA25 | pCRISPR-cBEST with sgRNA25 | This work |
| pcBEST-sgRNA26 | pCRISPR-cBEST with sgRNA26 | This work |
| pcBEST-sgRNA34 | pCRISPR-cBEST with sgRNA34 | This work |
| pcBEST-sgRNA92 | pCRISPR-cBEST with sgRNA92 | This work |
| pcBEST-sgRNA104 | pCRISPR-cBEST with sgRNA104 | This work |
| pcBEST-*upp* | pCRISPR-cBEST with  *upp* counterselection marker | This work |
| pCas9-*upp* | Derived from pcBEST with deletion of the cytidine deaminase editor and uracil glycosylase inhibitor, replaced the nCas9 (D10A) protein gene with the Cas9 protein gene, containing *upp* counterselection marker | This work |
| pnCas9-*upp* | Derived from pcBEST with deletion of the cytidine deaminase editor and uracil glycosylase inhibitor, retaining the nCas9 (D10A) protein gene and containing *upp* counterselection marker | This work |
| pCas9-*upp-endT-*sgRNA78 | pCas9-*upp* with two HAs flanking *endT*, sgRNA78, Cas9 protein | This work |
| pnCas9-*upp-endT-*sgRNA78 | pCas9-*upp* with two HAs flanking *endT*, sgRNA78, nCas9 protein | This work |
| pCas9-*upp-endT-*sgRNA123 | pCas9-*upp* with two HAs flanking *endT*, sgRNA123, Cas9 protein | This work |
| pnCas9-*upp-endT-*sgRNA123 | pCas9-*upp* with two HAs flanking *endT*, sgRNA123, nCas9 protein | This work |
| pCas9-*upp-endT-*sgRNA275 | pCas9-*upp* with two HAs flanking *endT*, sgRNA275, Cas9 protein | This work |
| pnCas9-*upp-endT-*sgRNA275 | pCas9-*upp* with two HAs flanking *endT*, sgRNA275, nCas9 protein | This work |
| pCas9-*upp-nrps1-*sgRNA449 | pCas9-*upp* with two HAs flanking *endT*, sgRNA449, Cas9 protein | This work |
| pCas9-*upp-nrps1-*sgRNA462 | pCas9-*upp* with two HAs flanking *endT*, sgRNA462, Cas9 protein | This work |
| pCas9-*upp-nrps1-*sgRNA841 | pCas9-*upp* with two HAs flanking *endT*, sgRNA841, Cas9 protein | This work |
|  |  |  |

**Table S3** The primers used in this study

| **Primers** | **Sequences (5’ to 3’)** | **Functions** |
| --- | --- | --- |
| *nrps*2-LF | GGATCCGACGGATACGGCTTCGCCCT | Amplification of the homologous arms of *nrps2* |
| *nrps2-*LR | TCTAGAGAGCGGGCGTACACCGAGAA |  |
| *nrps2-*RF | TCTAGACGACGAACTCGGCGTCGAAC |  |
| *nrps2-*RR | GAATTCAGGACCTCGTACCAGACATC |  |
| *nrps2tsr*F | **GGTGTACGCCCGCTCTCTAGA**TGATCAAGGCGAATACTTCATATGG | Amplification of the tsr gene between the homologous arms of *nrps2* |
| *nrps2tsr*R | **ACGCCGAGTTCGTCGTCTAGA**CAACGAGACGATGAAGCCGT |  |
| *nrps2*OUT-F | ACCCGACCGGCACTTCTGGT | Validation Primers on both sides of the *nrps2* homology arm for amplification and sequencing |
| *nrps2*OUT-R | TGACCGCAGGCGATTTCATG |  |
| *nrps*3-LF | GGATCCCAGGTGCTCGGCGAGGGAGG | Amplification of homologous arms of *nrps3* |
| *nrps*3-LR | TCTAGAGGATCACCCGCGACGGAGT |  |
| *nrps*3-RF | TCTAGAGTTGGAGACCATGCCGGGCA |  |
| *nrps*3-RR | GAATTCCTGTTCGTGTCCGCCATGCG |  |
| *nrps3tsrF* | **CTCGCCGAGCACCTGTCTAGA**TGATCAAGGCGAATACTTCATATGG | Amplification of the *tsr* gene between the homologous arms of *nrps3* |
| *nrps3tsrR* | **GGCGGACACGAACAGTCTAGA**CAACGAGACGATGAAGCCGT |  |
| *nrps*3OUT-F | GGCCGTAGAGGGCTACGTCC | Validation Primers on both sides of the *nrps3* homology arm for amplification and sequencing |
| *nrps*3OUT-R | CTGGACATCGAAGGAGCCGT |  |
| *pks1-LF* | GGATCCGGAAGACCTGGGCGAGTCGG | Amplification of homologous arms of *pks1* |
| *pks1-LR* | TCTAGATCTGTACGCCTGCTTCCGCA |  |
| *pks1-RF* | TCTAGAAGATGTGGTCACCGTCGCGC |  |
| *pks1-RR* | GAATTCACCACCGCCGGTTCTGGAAC |  |
| *pks*1*tsr*F | **AAGCAGGCGTACAGATCTAGA**TGATCAAGGCGAATACTTCATATGG | Amplification of the *tsr* gene between the homologous arms of *pks1* |
| *pks*1*tsr*R | **ACGGTGACCACATCTTCTAGA**CAACGAGACGATGAAGCCGT |  |
| *pks*1OUT-F | CGATCCCGCCACGTAGTTGC | Validation Primers on both sides of the *pks1* homology arm for amplification and sequencing |
| *pks1*OUT-R | CCACCGATGTCGCAGCATGT |  |
| M*upp*-sgRNA01-AF | cggttggtaggatcgacggcCAGGACGTACACCTGACGTCgttttagagctagaaatagc | Annealing to form dsDNA for sgRNA insertion, for mutations in the *upp* gene |
| M*upp*-sgRNA01-AR | gctatttctagctctaaaacGACGTCAGGTGTACGTCCTGgccgtcgatcctaccaaccg |  |
| M*upp*-sgRNA18-AF | cggttggtaggatcgacggcGAGCAGGGCTACATCGTGCCgttttagagctagaaatagc |  |
| M*upp*-sgRNA18-AR | gctatttctagctctaaaacGGCACGATGTAGCCCTGCTCgccgtcgatcctaccaaccg |  |
| M*upp*-sgRNA25-AF | cggttggtaggatcgacggcATCCAGGAGCTGATCAGGCGgttttagagctagaaatagc |  |
| M*upp*-sgRNA25-AR | gctatttctagctctaaaacCGCCTGATCAGCTCCTGGATgccgtcgatcctaccaaccg |  |
| M*upp*-sgRNA26-AF | cggttggtaggatcgacggcCGAGCAGGGCTACATCGTGCgttttagagctagaaatagc |  |
| M*upp*-sgRNA26-AR | gctatttctagctctaaaacGCACGATGTAGCCCTGCTCGgccgtcgatcctaccaaccg |  |
| M*upp*-sgRNA34-AF | cggttggtaggatcgacggcCGGACGTCAGGTGTACGTCCgttttagagctagaaatagc |  |
| M*upp*-sgRNA34-AR | gctatttctagctctaaaacGGACGTACACCTGACGTCCGgccgtcgatcctaccaaccg |  |
| M*upp*-sgRNA92-AF | cggttggtaggatcgacggcCGGCGATCCAGGAGCTGATCgttttagagctagaaatagc |  |
| M*upp*-sgRNA92-AR | gctatttctagctctaaaacGATCAGCTCCTGGATCGCCGgccgtcgatcctaccaaccg |  |
| M*upp*-sgRNA104-AF | cggttggtaggatcgacggcACATCCAGACGCCGGTGGAGgttttagagctagaaatagc |  |
| M*upp*-sgRNA104-AR | gctatttctagctctaaaacCTCCACCGGCGTCTGGATGTgccgtcgatcctaccaaccg |  |
| cb-mcsF | GAGCGTCGATTTTTGTGATGC | Sequencing primers for the sgRNA insertion of pcBEST, pCas9/nCas9-*upp* |
| cb-mcsR | GATCGAGTGCCGGTCGGTGT |  |
| Tesupp-F | CGTCTCCACGTCGTCGACCACC | Amplification of *upp* gene fragments and sequencing primers |
| Tesupp-R | CTCCGCCGCCCCGTACATCC |  |
| Pfd-uF | CCCGGGTACCGAGCTCGAATTCAAAATCTCCAAAAAAAAAGGCTCCAAAAGGAGCCTTTAATTGTATCGGTCAGGCGCCATTCGCCATT | Annealing to form dsDNA for fd terminator insertion |
| Pfd-uR | AATGGCGAATGGCGCCTGACCGATACAATTAAAGGCTCCTTTTGGAGCCTTTTTTTTTGGAGATTTTGAATTCGAGCTCGGTACCCGGG |  |
| Permupp-F | CAGGCGCCATTCGCCATTCAGGCTG | Amplification of the *upp* counterselection marker for insertion into the pcBEST vector |
| Permupp-R | CTTTAGATCTGGGGAATTACTGCTGAGGGCGGACGAACC |  |
| Pc-upp-tF | CAGGACTCCAACGGCGAGAACAAG | Sequencing primers for the *upp* insertion |
| Pc-upp-tR | GGCGCAACTATCGGTATCAAGCTG |  |
| PACV_Cas9-F | CGTCAGAGAAGGGAGCGGACATATGGACAAGAAGTACTCCATCGGCCTC**GAC**ATCGGC | Amplification of Cas9/nCas9 gene fragment from pcBEST |
| PACV_nCas9-F | CGTCAGAGAAGGGAGCGGACATATGGACAAGAAGTACTCCATCGGCCTC**GCG**ATCGGC |  |
| PACV_xCas9-R | GCCTTTTTTTTTGGAGATTTTGAATTCTCAGTCGCCGCCGAGCTGGGACAG |  |
| P_te-xCas9-F | GCGACAAGTTGCTGCGATTCTCAC | Sequencing primers for the Cas9/nCas9 gene fragment of the pCas9/nCas9-*upp* vector |
| P_te-xCas9-R | GATCGGACGGATTTGCGATGGTG |  |
| P_xcap_xbaI-t5620up-F | CAAGCTTGGGCTGCAGGTCGACTCTAGAGTCCTCCCCCTCGAAGGCCCT | Amplification of homologous arms of *endT* gene |
| P_xca-t5620up-R | CTGTGTGTCGTCGGGGCGTCGCCCGCCTCTCCCTCCTCGTCGT |  |
| P_xca-t5620down-F | ACGACGAGGAGGGAGAGGCGGGCGACGCCCCGACGACACACAGCG |  |
| P_xcap_xbaI-t5620down-R | TCCCAACAGTTGCGCAGCTCTAGAAGCAGCGAGGTGAGGGCCT |  |
| xcas-5620-sgRNA78-F | cggttggtaggatcgacggcTCATGAGCACCGGTTCGCCCgttttagagctagaaatagc | Annealing to form dsDNA for sgRNA insertion, for deletion of the *endT* gene |
| xcas-5620-sgRNA78-R | gctatttctagctctaaaacGGGCGAACCGGTGCTCATGAgccgtcgatcctaccaaccg |  |
| xcas-5620-sgRNA123-F | cggttggtaggatcgacggcTCTGGTGCAGGGTCCAGGCAgttttagagctagaaatagc |  |
| xcas-5620-sgRNA123-R | gctatttctagctctaaaacTGCCTGGACCCTGCACCAGAgccgtcgatcctaccaaccg |  |
| xcas-5620-sgRNA275-F | cggttggtaggatcgacggcCCCTGGATCTGGCGCCGTGCgttttagagctagaaatagc |  |
| xcas-5620-sgRNA275-R | gctatttctagctctaaaacGCACGGCGCCAGATCCAGGGgccgtcgatcctaccaaccg |  |
| xcasup-udF | GCATCGACCTGTCCCAGCT | Sequencing primers for the homologous arms of the pCas9/nCas9-*upp* vector |
| xcasup-udR2 | CTCCTTTCGCTGGCGTCAAC |  |
| Kno25620-cF | CGGCCACGAGAGGTAGAGCAT | Validation Primers on both sides of the *endT* homology arm for amplification and sequencing |
| Kno25620-cR | GCTCAGGGGTTCGAGCTGCT |  |
| Kno25620-cF2 | GGATGAAGAACAGCGGGATCAT |  |
| Kno25620-cR2 | GATCTCCACGTACTCGGCGAT |  |
| P_cap_xbaI-k01470-uF | CAAGCTTGGGCTGCAGGTCGACTCTAGAGGTCGCGGAGATCAGAGCGT | Amplification of homologous arms of *nrps1* |
| P_cap-k01470-uR | TGTGCCGTCGGCCCGGTCAAGCGCAAACCTCACGTGGC |  |
| P_cap-k01470-dF | GCCACGTGAGGTTTGCGCTTGACCGGGCCGACGGCACAC |  |
| P_cap_xbaI-k01470-dR | TCCCAACAGTTGCGCAGCTCTAGACAGGGCCAGGACGGCCTTGATGAGT |  |
| cas-01470sgRNA462-F | cggttggtaggatcgacggcCGTTGTACGTCTCGGCCAGGgttttagagctagaaatagc | Annealing to form dsDNA for sgRNA insertion, for deletion of *nrps1* |
| cas-01470sgRNA462-R | gctatttctagctctaaaacCCTGGCCGAGACGTACAACGgccgtcgatcctaccaaccg |  |
| cas-01470sgRNA841-F | cggttggtaggatcgacggcGTCCACGAAGAGGTCGAACTgttttagagctagaaatagc |  |
| cas-01470sgRNA841-R | gctatttctagctctaaaacAGTTCGACCTCTTCGTGGACgccgtcgatcctaccaaccg |  |
| cas-01470sgRNA449-F | cggttggtaggatcgacggcCATGCGGATCGCCGACGAACgttttagagctagaaatagc |  |
| cas-01470sgRNA449-R | gctatttctagctctaaaacGTTCGTCGGCGATCCGCATGgccgtcgatcctaccaaccg |  |
| T_Kno01470-in75-CF | GATGCGCATCTCGTGCACGT | Validation Primers on both sides of the *nrps1* homology arm for amplification and sequencing |
| T_Kno01470-in65-CR | GCGAGGTGCCTCCTCCAGGAT |  |
|  |  |  |

The restriction sites are underlined. The overlapped sequences used for one-step cloning are in bold or lowercase.

**Table S4** The BGCs predicted by antiSMASH in *S. fungicidicus*

| **Region** | **Type** | **From** | **To** | **Most similar known cluster** | **Product type** | **Similarity** |
| --- | --- | --- | --- | --- | --- | --- |
| Region 1 | transAT-PKS | 39,656 | 151,177 | dumulmycin/ shuangdaolide A, B, C, D | Polyketide | 85% |
| Region 2 | T1PKS, NRPS | 337,323 | 389,842 | antimycin | NRP+Polyketide | 100% |
| Region 3 | betalactone | 485,610 | 514,220 | - | - | - |
| Region 4 | NRPS,other | 1,386,130 | 1,465,246 | hormaomycin/hormaomycin A1, A2, A3, A4, A5, A6 | NRP:Cyclic depsipeptide | 100% |
| Region 5 | ectoine | 1,788,658 | 1,799,056 | ectoine | Other | 100% |
| Region 6 | HR-T2PKS, NRPS | 2,241,917 | 2,323,784 | WS9326B/WS9326A/WS9326G/WS9326F | NRP | 100% |
| Region 7 | melanin | 2,746,366 | 2,756,815 | istamycin | Saccharide | 4% |
| Region 8 | NI-siderophore | 2,823,588 | 2,853,360 | FW0622 | Other | 62% |
| Region 9 | lanthipeptide-class-i | 3,678,160 | 3,703,431 | - | - | - |
| Region 10 | terpene | 5,167,066 | 5,188,151 | albaflavenone | Terpene | 100% |
| Region 11 | NRPS | 5,628,022 | 5,721,406 | enduracidin | NRP | 54% |
| Region 12 | terpene | 5,762,228 | 5,784,384 | geosmin | Terpene | 100% |
| Region 13 | NI-siderophore | 5,905,150 | 5,936,236 | paulomycin | Other | 13% |
| Region 14 | terpene | 5,940,982 | 5,966,560 | isorenieratene | Terpene | 100% |
| Region 15 | NI-siderophore | 6,430,646 | 6,460,493 | kinamycin | Polyketide | 19% |
| Region 16 | hglE-KS, T1PKS | 6,629,134 | 6,683,214 | A33853 | Other | 60% |

**Table S5** The detailed information of deleted genomic regions and the constructed genome-reduced SF△*upp* mutant strains

| **Strain** | **Deletion regions** | **Detailed gene ID** | **Coding product** | **Deletion size (bp)** | **Deletion region(start-end)** |
| --- | --- | --- | --- | --- | --- |
| SF△*upp* (original strain) | |  |  |  |  |
| SF1 | Region1 (pks1) | CNQ36_00305 CNQ36_00310 CNQ36_00315 CNQ36_00320 | transAT-PKS | 54,430 | 76,747-131,177 |
| SF2 | Region4 (nrps2) | CNQ36_06355 CNQ36_06360 | NRPS+other | 26,088 | 1,406,130 -1,432,217 |
| SF3 | Region6 (nrps3) | CNQ36_10285 CNQ36_10290 CNQ36_10295 | NRPS+HR-T2PKS | 23,768 | 2,280,017-2,303,784 |
| SF4 | Strain derived from SF3 with the counter-selection marker removed | - | - | - | - |
| SF5 | Region4 (nrps2), Region6 (nrps3) | CNQ36_06355  CNQ36_06360  CNQ36_10285  CNQ36_10290 CNQ36_10295 | NRPS+other, NRPS+HR-T2PKS | 49,856 | 1,406,130-1,432,217,  2,280,017-2,303,784 |
| SF6 | Region2 (nrps1) | CNQ36_01470 | NRPS+T1PKS | 8,637 | 3,612,06-3,69,842 |
| SF7 | Region2 (nrps1), Region4 (nrps2), Region6 (nrps3) | CNQ36_06355  CNQ36_06360  CNQ36_10285  CNQ36_10290  CNQ36_10295  CNQ36_01470 | NRPS+T1PKS, NRPS+other, NRPS+HR-T2PKS | 58,493 | 361206-369842,  1406130 -1432217,  2280017-2303784 |

**Table S6** The sgRNAs for base mutations of *upp* gene predicted by CRISPy-web

| ID | Start | End | Strand | ORF | Sequence | PAM | C to T mutations | A to G mutations |
| --- | --- | --- | --- | --- | --- | --- | --- | --- |
| CY00000001 | 364 | 387 | -1 | CNQ36_16770 | CAGGACGTACACCTGACGTC | CGG | V128I | Y127H, V128A |
| CY00000018 | 570 | 593 | 1 | CNQ36_16770 | GAGCAGGGCTACATCGTGCC | GGG | Q192* | Q192R |
| CY00000025 | 426 | 449 | 1 | CNQ36_16770 | ATCCAGGAGCTGATCAGGCG | CGG | Q144* | Q144R, E145G |
| CY00000026 | 569 | 592 | 1 | CNQ36_16770 | CGAGCAGGGCTACATCGTGC | CGG | Q192* | Q192R |
| CY00000034 | 365 | 388 | 1 | CNQ36_16770 | CGGACGTCAGGTGTACGTCC | TGG | R124C, Q125* | Q125R |
| CY00000092 | 421 | 444 | 1 | CNQ36_16770 | CGGCGATCCAGGAGCTGATC | AGG | A142V, Q144* | I143V, Q144R |
| CY00000104 | 157 | 180 | 1 | CNQ36_16770 | ACATCCAGACGCCGGTGGAG | CGG | Q55*, T56M | Q55R, T56A |

**Table S7** The sgRNAs for Cas9 cleavage predicted by CRISPy-web

| ID | Start | End | Strand | ORF | Sequence | PAM |
| --- | --- | --- | --- | --- | --- | --- |
| CY00000078 | 77 | 100 | -1 | CNQ36_25620 | TCATGAGCACCGGTTCGCCC | CGG |
| CY00000123 | 122 | 145 | -1 | CNQ36_25620 | TCTGGTGCAGGGTCCAGGCA | TGG |
| CY00000275 | 274 | 297 | 1 | CNQ36_25620 | CCCTGGATCTGGCGCCGTGC | CGG |
| CY00000462 | 461 | 484 | -1 | CNQ36_01470 | CGTTGTACGTCTCGGCCAGG | CGG |
| CY00000842 | 4336 | 4359 | -1 | CNQ36_01470 | GTCCACGAAGAGGTCGAACT | TGG |
| CY00000449 | 6086 | 6109 | 1 | CNQ36_01470 | CATGCGGATCGCCGACGAAC | AGG |

**Table S8** Expression levels (TPM) of target gene clusters in *Streptomyces fungicidicus* TXX3120 at different time points

| Known cluster | Short name | Gene ID | Expression level (TPM) | | | | | | | |  |
| --- | --- | --- | --- | --- | --- | --- | --- | --- | --- | --- | --- |
|  |  |  | 32 h | | 48 h | | 72 h | | 144 h | |  |
|  |  |  | R2-2 | R2-1 | R4-1 | R4-2 | R5-1 | R5-2 | R6-1 | R6-2 | |
| dumulmycin/ shuangdaolide A, B, C, D | *pks1* | CNQ36_00305 | 4.170691 | 6.481334 | 14.267215 | 8.492693 | 64.184952 | 69.843224 | 2.221483 | 0 | |
|  |  | CNQ36_00310 | 1.394162 | 4.42735 | 0 | 10.591638 | 48.122227 | 60.975597 | 2.818361 | 3.035259 | |
|  |  | CNQ36_00315 | 3.999343 | 5.202803 | 11.444008 | 8.555215 | 8.916114 | 7.895299 | 1.654773 | 1.830173 | |
|  |  | CNQ36_00320 | 3.312946 | 7.183742 | 12.070375 | 12.32874 | 62.897141 | 67.212273 | 1.102811 | 1.480598 | |
| hormaomycin/  hormaomycin A1, A2, A3, A4, A5, A6 | *nrps2* | CNQ36_06355 | 1.959098 | 3.142164 | 7.836471 | 13.956631 | 13.959468 | 49.75668 | 3.95974 | 3.748045 | |
|  |  | CNQ36_06360 | 1.420586 | 5.727747 | 19.251627 | 13.352449 | 39.7132 | 52.599556 | 2.170079 | 2.674196 | |
| WS9326B/WS9326A/WS9326G/WS9326F | *nrps3* | CNQ36_10285 | 1.532555 | 4.337879 | 1.637336 | 2.31424 | 84.842545 | 94.867973 | 3.113027 | 1.30274 | |
|  |  | CNQ36_10290 | 0 | 4.127246 | 2.807861 | 3.122554 | 0 | 111.78215 | 1.105379 | 0 | |
|  |  | CNQ36_10295 | 1.072692 | 0 | 2.042523 | 2.254362 | 75.159958 | 95.983192 | 0.478035 | 1.477065 | |
| antimycin | *nrps1* | CNQ36_01470 | 1.360598 | 0 | 1.470134 | 3.270641 | 73.55394 | 82.338837 | 0.331936 | 1.934321 | |
| enduracidin | *endA* | CNQ36_25630 | 6.082912 | 2.370303 | 176.31923 | 190.34283 | 150.20917 | 263.65173 | 30.931007 | 32.638607 | |
|  | *endB* | CNQ36_25635 | 5.088231 | 0 | 200.56357 | 37.502777 | 133.42116 | 285.237 | 75.805405 | 7.46666 | |
|  | *endC* | CNQ36_25640 | 1.490336 | 12.016882 | 44.106602 | 39.69487 | 47.955727 | 2.522943 | 22.811783 | 8.185555 | |
|  | *endD* | CNQ36_25655 | 2.910178 | 6.313008 | 145.17276 | 117.1247 | 106.25691 | 193.23344 | 44.241608 | 57.526516 | |


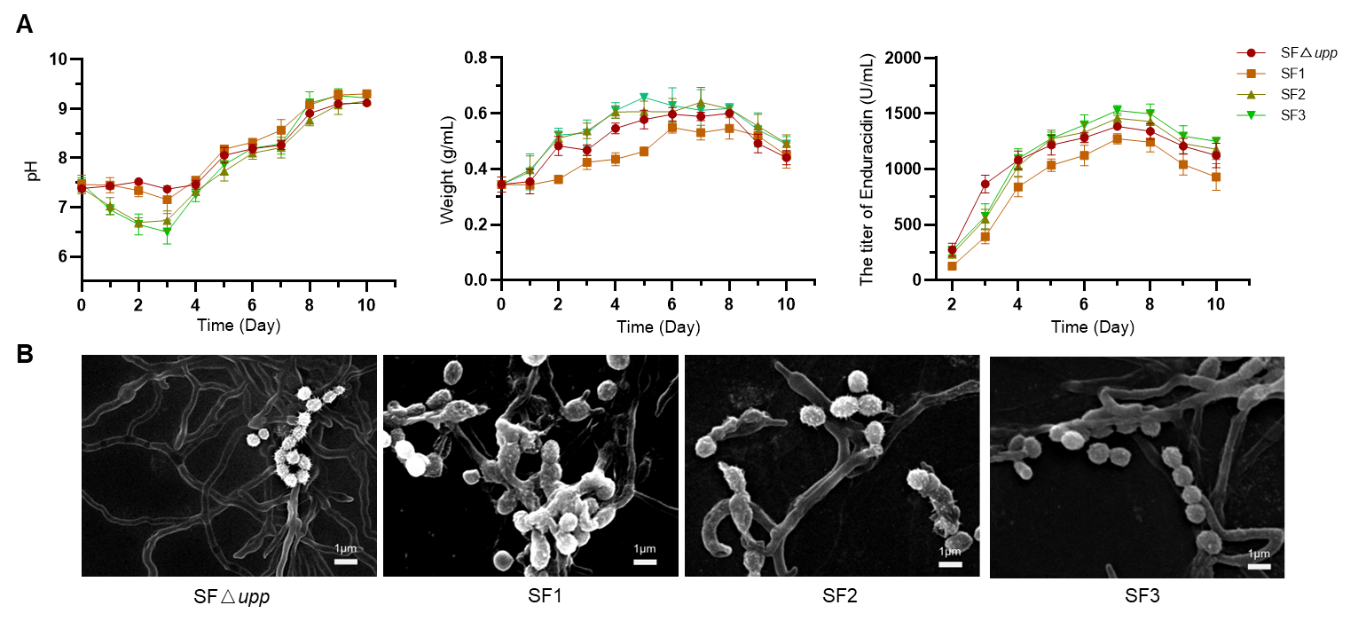


**Figure S1** Characterization of SF△*upp* and the mutants SF1, SF2, SF3. **(A)** The pH variation, growth profiles, and the enduracidin titer of the mutants and SF△*upp,* respectively*.* **(B)** Cell morphology determination by using SEM of the mutants and SF△*upp*.


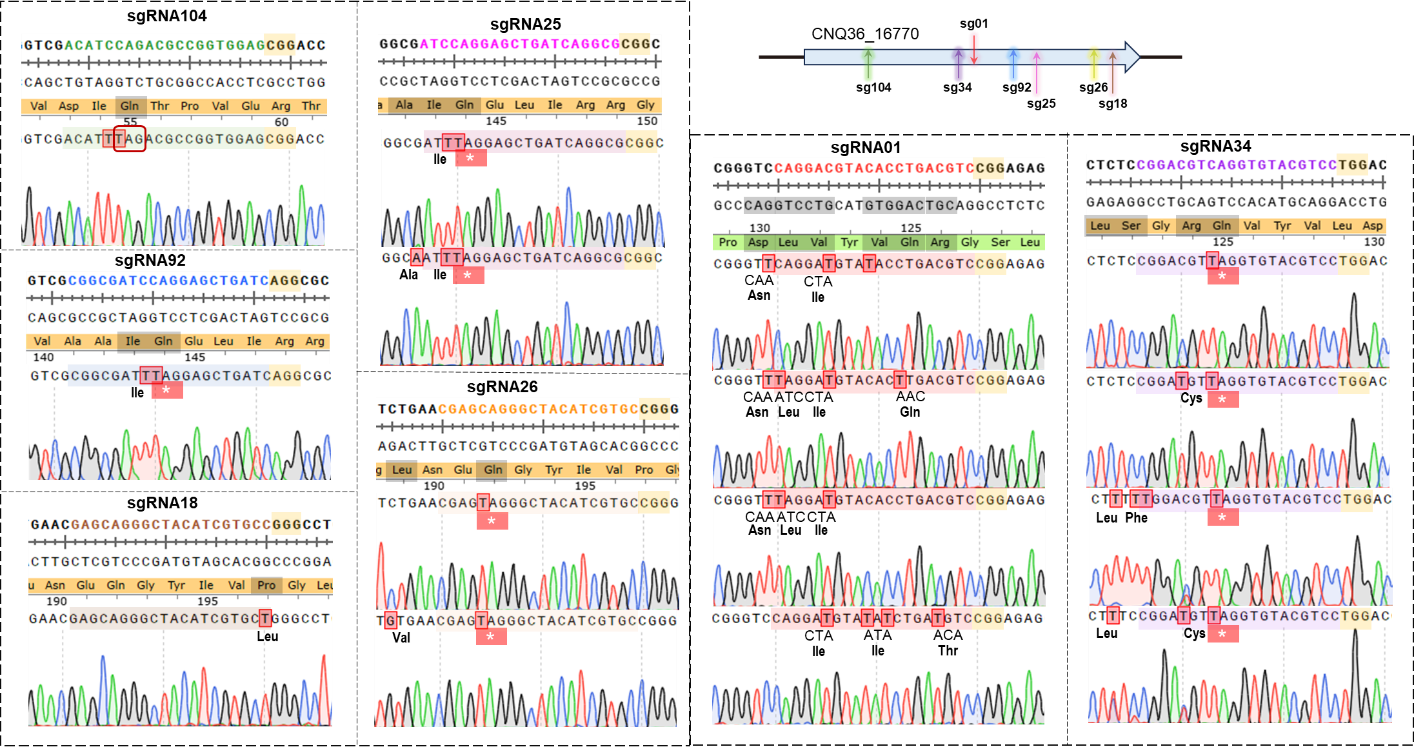


**Figure S2** The sequencing results of base editing events in mutants were obtained using the CRISPR/cBEST system with different sgRNAs.


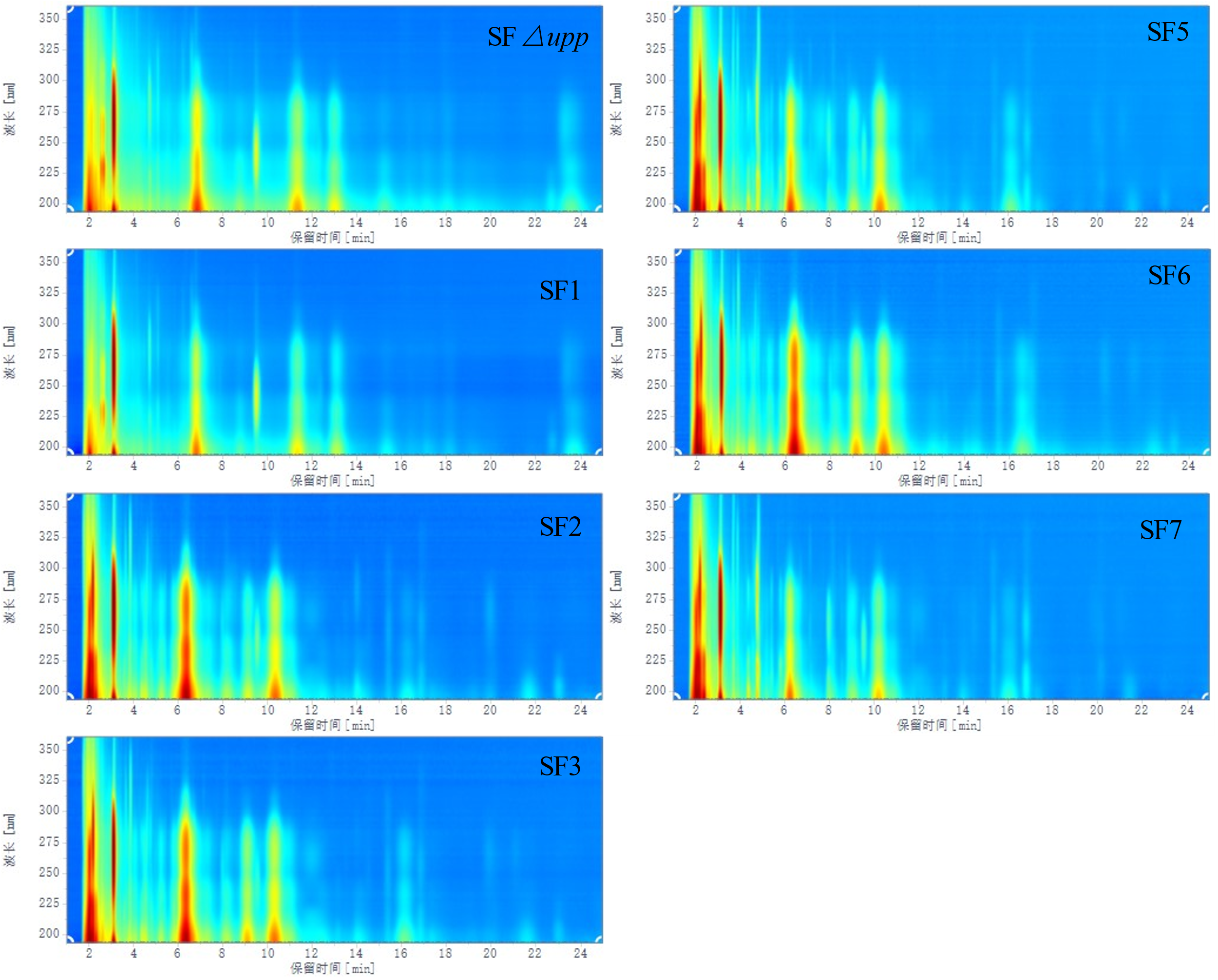


**Figure S3** Metabolite profile analysis based on iso-absorbance plot. Iso-absorptive curves of the fermentation extracts from mutant strains were analyzed using a diode array detector.


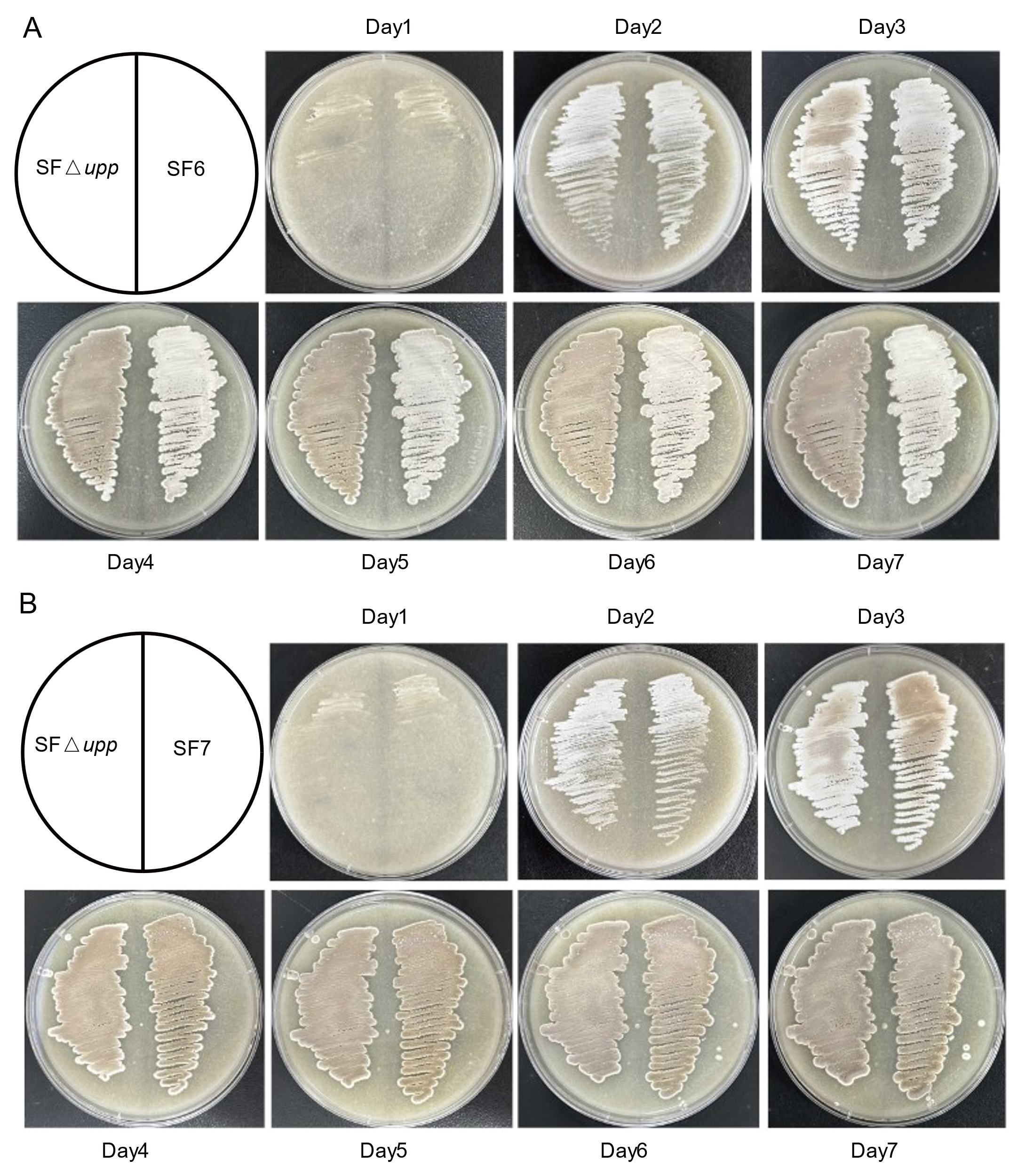


**Figure S4** Colony morphology of growth mutants compared to SF△*upp* in MS medium. **(A)** The growth of bacterial colonies of SF6. **(B)** The growth of bacterial colonies of SF7.


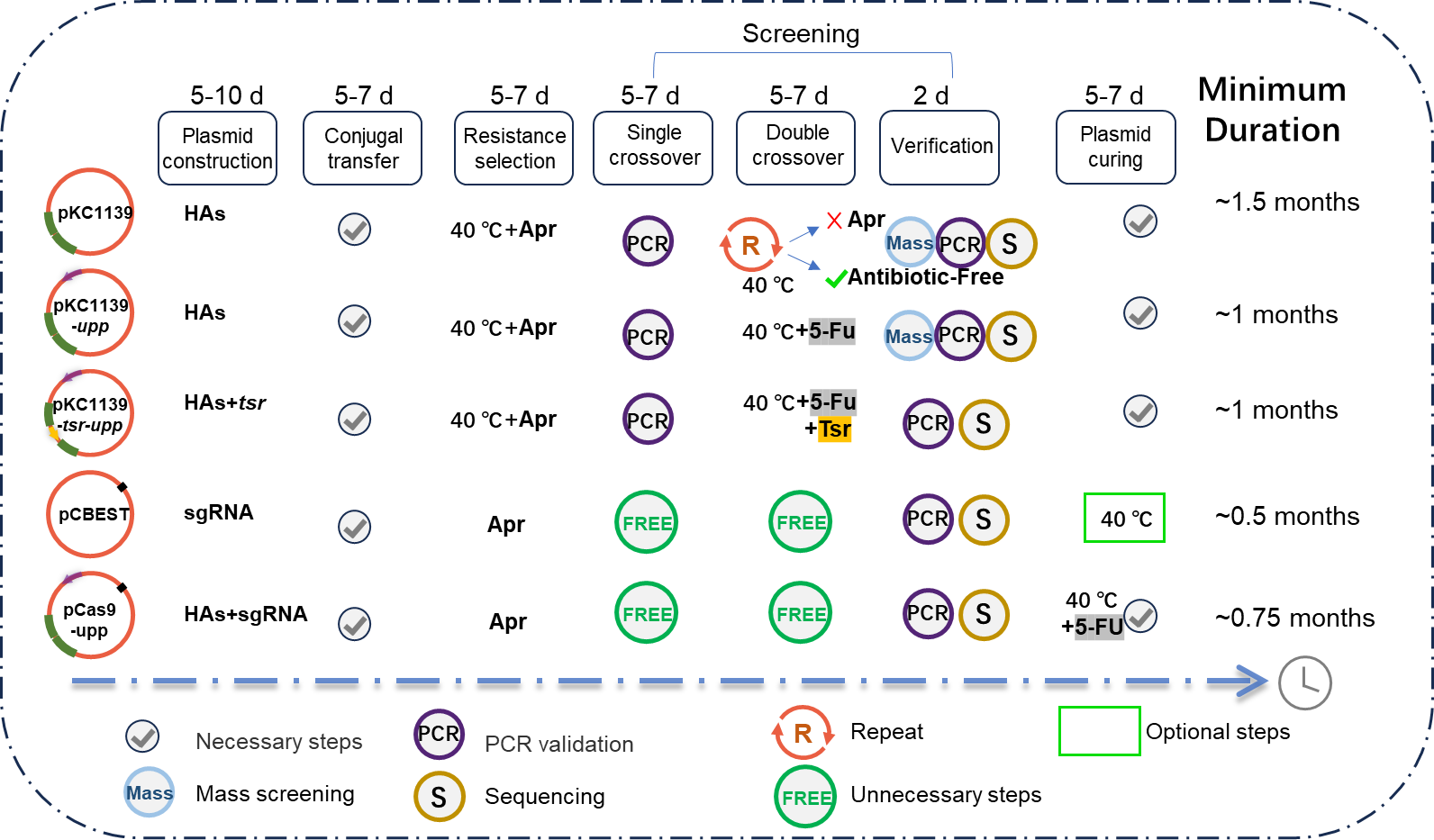


**Figure S5** An overview of experimental steps required by different plasmids. The diagram shows the progress of using different type of plasmids to complete the desired genetic manipulation in *Streptomyces fungicidicus* TXX3120.


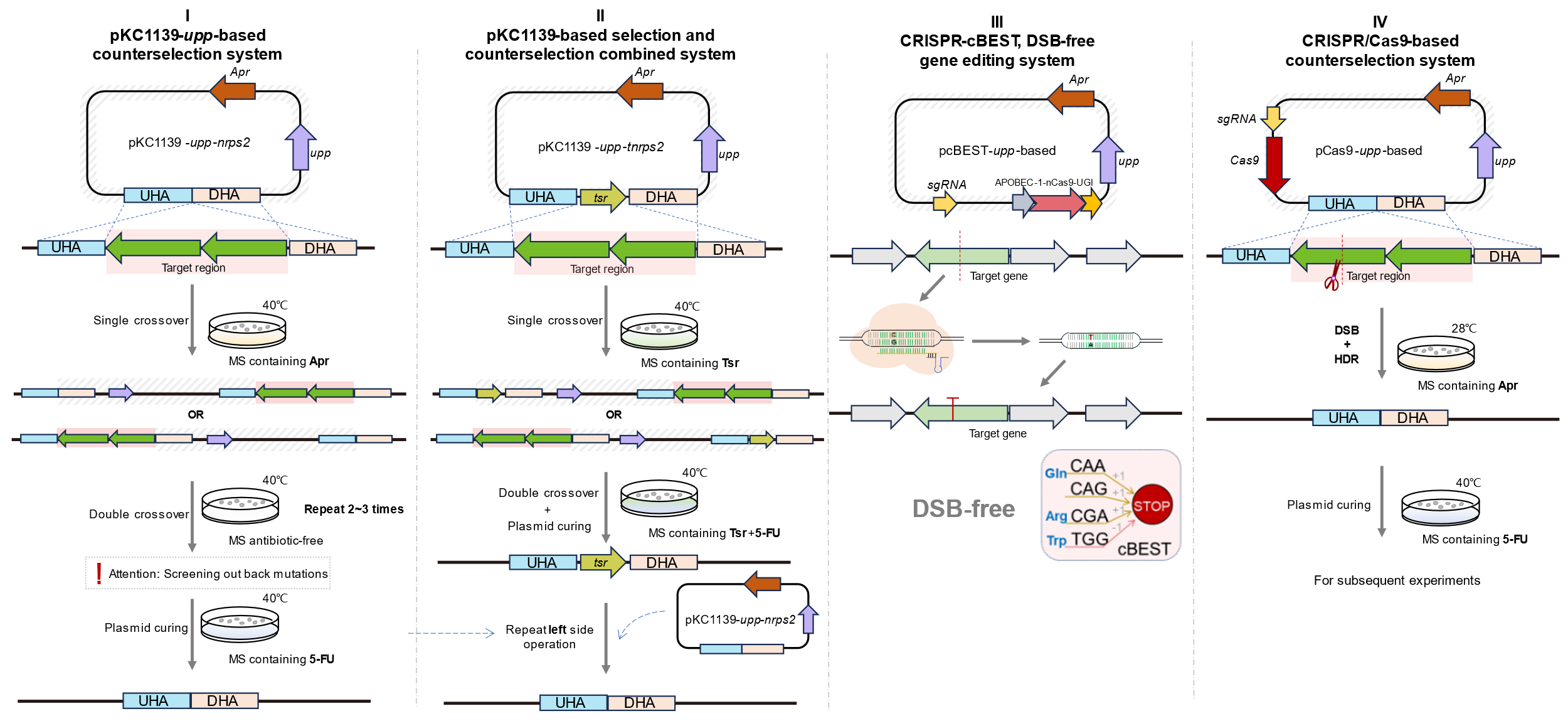


**Figure S6** The schematic diagram of detailed experimental steps required by different type of plasmids.


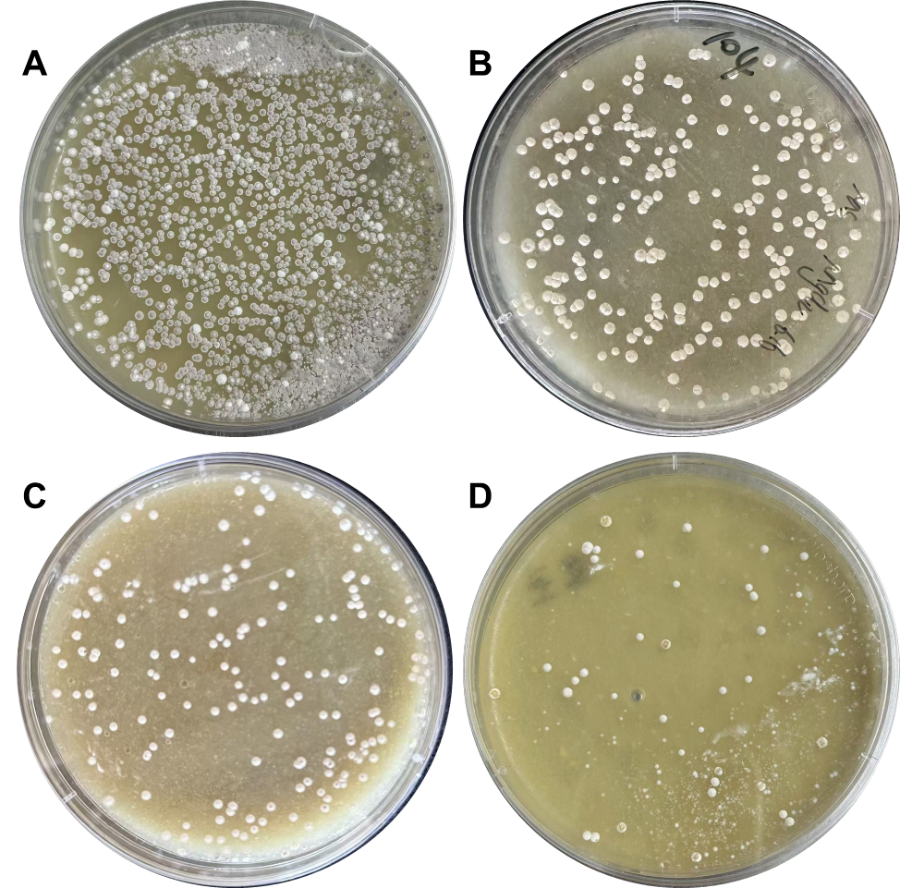


**Figure S7** The conjugal transfer plates of *Streptomyces fungicidicus* TXX3120 using different plasmids. (A) The higher number of transformants grown using the pKC1139-*upp*-based plasmids on conjugal transfer plate. (B) The conjugal transfer plate using the pcBEST-based plasmid. (C) The conjugal transfer plate using the pnCas9-*upp*-based plasmid. (C) The conjugal transfer plate using the pCas9-*upp*-based plasmid. The low number of transformants may be due to the toxicity of Cas9 protein.
